# Assembly, Annotation, and Comparative Analysis of Mitochondrial Genomes in *Trichoderma*

**DOI:** 10.3390/ijms252212140

**Published:** 2024-11-12

**Authors:** Xiaoting Wang, Zhiyin Wang, Fanxing Yang, Runmao Lin, Tong Liu

**Affiliations:** Key Laboratory of Green Prevention and Control of Tropical Plant Diseases and Pests, Ministry of Education, School of Tropical Agriculture and Forestry, School of Breeding and Multiplication (Sanya Institute of Breeding and Multiplication), Hainan University, Sanya 572025, China; 22210904000011@hainanu.edu.cn (X.W.); 22220951320020@hainanu.edu.cn (Z.W.); 22220951320039@hainanu.edu.cn (F.Y.)

**Keywords:** *Trichoderma*, mitochondrial genome, genetic variation, phylogeny, positive selection

## Abstract

*Trichoderma* is a widely studied ascomycete fungal genus, including more than 400 species. However, genetic information on *Trichoderma* is limited, with most species reporting only DNA barcodes. Mitochondria possess their own distinct DNA that plays a pivotal role in molecular function and evolution. Here, we report 42 novel mitochondrial genomes (mitogenomes) combined with 18 published mitogenomes of *Trichoderma*. These circular mitogenomes exhibit sizes of 26,276–94,608 bp, typically comprising 15 core protein-coding genes (PCGs), 2 rRNAs, and 16–30 tRNAs; however, the number of endonucleases and hypothetical proteins encoded in the introns of PCGs increases with genome size enlargement. According to the result of phylogenetic analysis of the whole mitogenome, these strains diverged into six distinct evolutionary branches, supported by the phylogeny based on 2830 single-copy nuclear genes. Comparative analysis revealed that dynamic *Trichoderma* mitogenomes exhibited variations in genome size, gene number, GC content, tRNA copy, and intron across different branches. We identified three mutation hotspots near the regions encoding *nad3*, *cox2*, and *nad5* that caused major changes in the mitogenomes. Evolutionary analysis revealed that *atp9*, *cob*, *nad4L*, *nad5*, and *rps3* have been influenced by positive selection during evolution. This study provides a valuable resource for exploring the important roles of the genetic and evolutionary dynamics of *Trichoderma* mitogenome in the adaptive evolution of biocontrol fungi.

## 1. Introduction

*Trichoderma* (Hypocreales) is a genus of biocontrol fungi that is ubiquitous in ecological environments. It has the capacity to control pathogenic microorganisms, nematodes, and insects, and has been used extensively in the fields of biological control and the development of biocontrol agents [1,2,3]. *Trichoderma* is rich in species diversity and is widely distributed, consisting of more than 400 species (of which 375 species have been effectively named by 2020) [4], and can be used as an ideal material for evolutionary research. However, genetic information on *Trichoderma* is limited, with most species reporting only DNA barcodes. The DNA barcodes ITS, *rpb2*, and *tef1* are frequently used for the molecular identification of *Trichoderma* spp. [4]. A total of 357 species with unambiguous identification have been reported with DNA barcodes available in the National Center for Biotechnology Information (NCBI) nucleotide database (August 2022). Of these, 243 species (64.8%) have been reported with all three DNA barcode genes (ITS, *tef1*, and *rpb2*). DNA barcodes and a lack of genomic resources have hindered the understanding and species identification of *Trichoderma*.

Genetic and comparative genomic studies have shown that *Trichoderma* spp. continually reconstruct their genomes to enhance their ability to rapidly colonize and compete for nutrients and space in new habitats [5,6,7]. Intensive studies on the evolution of *Trichoderma* have revealed the existence of large-scale horizontal gene transfers in its genome [8] and that it gained a large number of genes during its evolution, as well as the existence of gene loss events, which contributed to the genomic diversity observed in *Trichoderma* spp. [7].

Previous studies on the evolution of *Trichoderma* have primarily been conducted at the nuclear genome level. Compared to nuclear genome studies, mitogenome data are easier to obtain and are more conducive to analyzing the genetics and evolution of *Trichoderma*, promoting our understanding of the diversity of *Trichoderma*. In 2020, Kwak et al. conducted a comparative analysis of the *Trichoderma* mitogenome from the perspective of mitochondrial evolution for the first time [9]. In response to intense selective pressures, organisms may have evolved adaptations that enable their survival under specific ecological conditions. This process involves site-specific amino acid substitutions that affect the protein structure and function [10]. In 2021, Kwak et al. proposed that the isolation of *Trichoderma* from a range of different environments (including soil, wood, living plants, and fungi), particularly those containing oxygen, was associated with the adaptive diversity of *Trichoderma* mitochondria [11]. Consequently, an analysis of the *Trichoderma* mitogenome and its associated diversity characteristics will facilitate an understanding of the evolutionary history of *Trichoderma* species.

Mitochondria are vital organelles that are pervasively distributed within eukaryotic cells and exert a pivotal influence on energy production, cellular respiration, cellular aging, and apoptosis [12]. Mitochondrial DNA (mtDNA) is a form of genetic information independent of the nuclear genome. It is characterized by its uniqueness, stability, and rapid evolution. Consequently, it is an ideal tool for examining the genetic diversity within fungal populations and identifying fungal species or isolates at the genetic level [13,14,15]. The fundamental structure of the fungal mitogenome includes protein-coding genes, transfer RNA (tRNA), ribosomal RNA (rRNA), introns, homing endonucleases, and other genes [16]. The mitogenome is characterized by its simple structure, small molecular weight, unique and stable sequences, and rapid mutation rate [17]. It has a high AT content, high copy number, low methylation levels, and low recombination rate [18,19]. The gene-coding region is relatively conserved, the intergenic region exhibits a high degree of variability, and the introns of the gene lead to high genomic polymorphisms [20]. Mitogenome sequences contain important genetic inforfmation on species differentiation and are widely used to study phylogenetic relationships among species [21,22]. By studying the structure of mitogenomes, we can determine the characteristics of ancestral mitogenomes and explore their evolution and adaptive evolution [23,24].

Studying the variations in the *Trichoderma* mitogenome in gene composition, structure, order, and other aspects can provide valuable insights into the evolution of species [9,11]. However, the available *Trichoderma* mitogenome data is limited, with only 18 complete *Trichoderma* mitogenomes reported in the NCBI up to October 2023. This limits our understanding of these fungal species. Therefore, more *Trichoderma* mitogenomes are required to facilitate an in-depth exploration of the genetic variation and evolution of *Trichoderma* species.

In this study, we report 42 *Trichoderma* mitogenomes combined with 18 published *Trichoderma* mitogenomes, yielding a total of 60 mitogenomes, and adding new molecular data for *Trichoderma*. The composition and structure of the *Trichoderma* mitogenome were analyzed and a phylogenetic tree was constructed using the complete mitogenome sequence. Subsequently, a comparative analysis was conducted on the genome size and gene number, GC content, arrangement and copy number of tRNA, and intron number of *Trichoderma* mitogenomes to explore the genetic variation in *Trichoderma* mitogenomes. In addition, a selection pressure analysis was performed on 15 conserved PCGs within the *Trichoderma* mitogenome. This study facilitates the understanding of the evolution of *Trichoderma* and contributes to revealing the significant role of vital genes in the adaptive evolution of *Trichoderma*.

## 2. Results

### 2.1. Identification of Trichoderma spp. Using Phylogenetic Analysis of ITS, tef1, and rpb2 Genes

**Figure 1:**
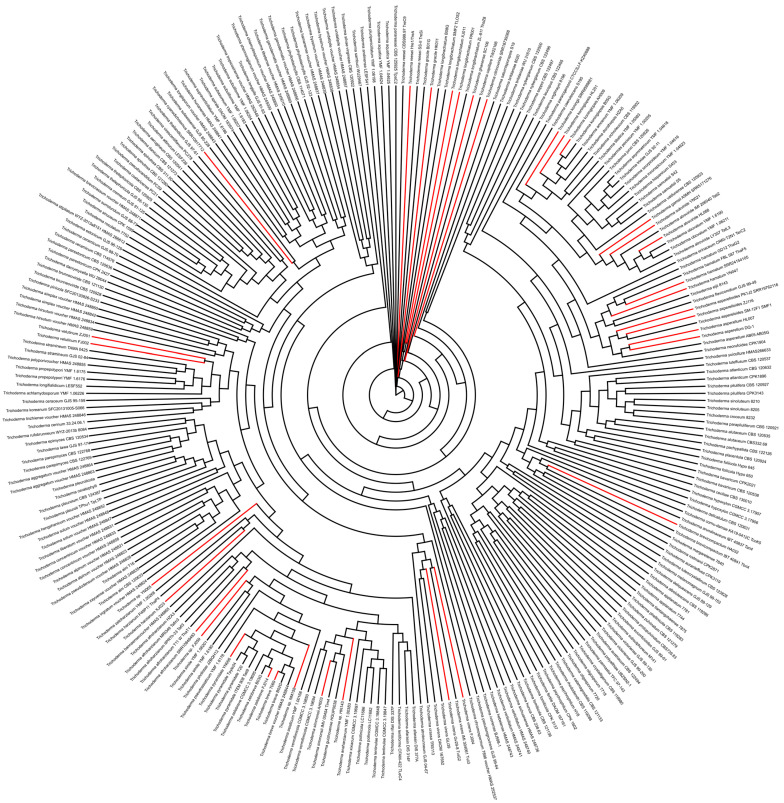
The phylogenetic tree of 194 *Trichoderma* species (273 strains). Gene sequences of ITS, *tef1*, and *rpb2* were used to construct the phylogenetic tree based on the GTR + I + G model using Raxml-ng with a bootstrap value of 500. The best model for phylogenetic analysis was detected using jModelTest (version 2.1.10). The strains whose mitogenomes were newly reported in this study are shown in red.

We collected the barcode sequences of ITS, *tef1*, and *rpb2* from 194 *Trichoderma* spp. (273 strains) for phylogenetic analysis, including sequences available in the NCBI Nucleotide database or inferred from genome assemblies (Supplementary Table S5, 01.seq.EF1.fa; 01.seq.ITS.fa; 01.seq.RPB2.fa [25]). Based on the phylogenetic tree (Figure 1), we identified new strains and reported strains that formed independent branches, such as T069, B02G, and *T. breve* voucher HMAS248844 formed one branch; FJ004, *T. virens* IMI 304061 Tvii3, *T. virens* Gv29-8 TviG2, *T. virens* GLi 39, and *T. virens* DAOM 167652 formed another branch, suggesting that T069 and B02G are *T. breve* strains and FJ004 is *T. virens*.

Sequence alignment between T069 and *T. breve* voucher HMAS248844 showed sequence identities of 0.99, 1.00, and 0.99 for ITS, *tef1*, and *rpb2*, respectively (Figures S1–S3). Based on the sequence alignment and phylogenetic analyses, we identified T069 as a *T. breve* strain. Similarly, for strains that were most closely related to lineages of known species, such as YN006, parallel to one branch of three *T. pyramidale* strains (ITEM 908 Tati9, Tpyle24, and T20), we analyzed the identity of gene sequences between YN006 and ITEM 908 Tati9, with 0.99 for ITS, 1 for *tef1* and 0.98 for *rpb2*, suggesting that YN006 was a *T. pyramidale* strain. However, the current evidence cannot identify the species names of the five strains (YN065, FJ059, SRR12137155, HN143, and NM158) because of the high level of sequence identity between each of the five strains and several reported species (Supplementary Table S5). Finally, based on the phylogenetic relationships and sequence alignments, we identified or confirmed the species names of 37 fungal strains, excluding five strains (YN065, FJ059, SRR12137155, HN143, and NM158).

### 2.2. Trichoderma Mitogenome Organization and Features

**Figure 2:**
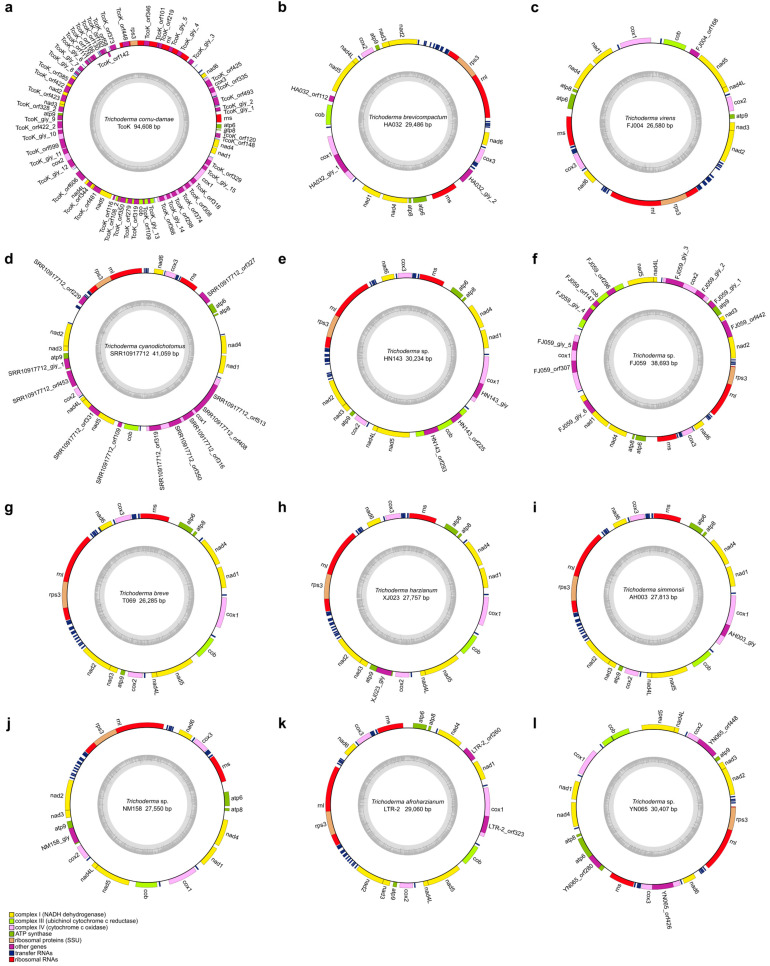
Mitogenome map of *T*. *cornu-damae* TcoK (**a**), *T*. *brevicompactum* HA032 (**b**), *T*. *virens* FJ004 (**c**), *T. cyanodichotomus* SRR10917712 (**d**), *Trichoderma* sp. HN143 (**e**), *Trichoderma* sp. FJ059 (**f**), *T*. *breve* T069 (**g**), *T. harzianum* XJ023 (**h**), *T. simmonsii* AH003 (**i**), *Trichoderma* sp. NM158 (**j**), *T*. *afroharzianum* LTR-2 (**k**), and *Trichoderma* sp. YN065 (**l**). The outermost layer lists the gene composition of 15 core PCGs, 2 rRNA genes, and tRNA genes (represented by filled boxes in different colors).

**Figure 3:**
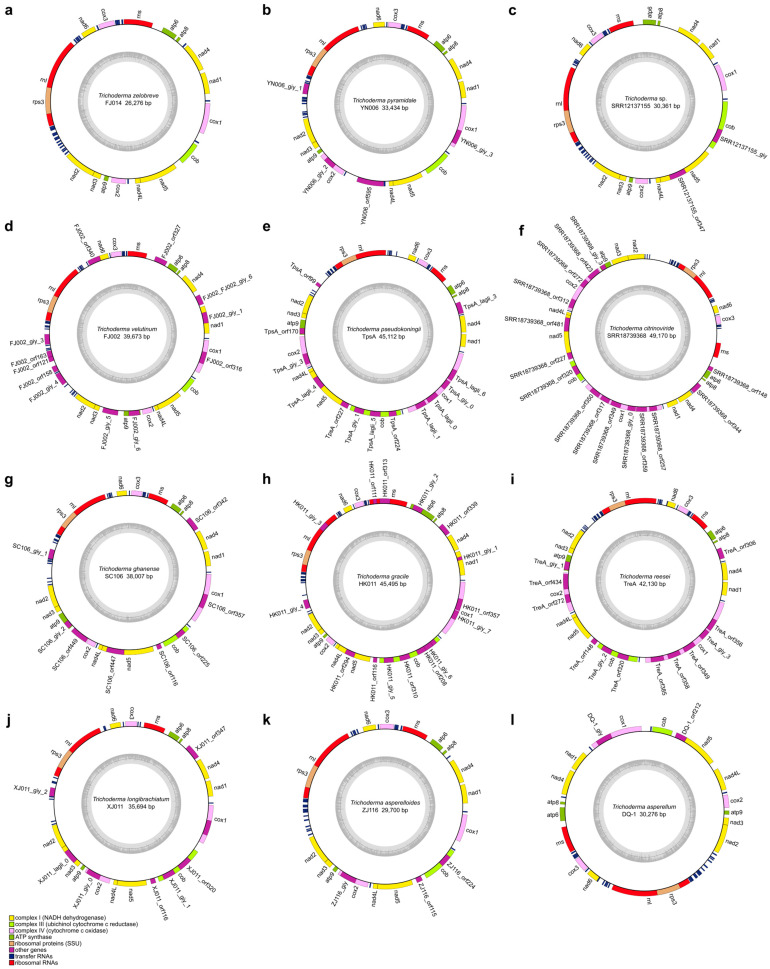
Mitogenome map of *T. zelobreve* FJ014 (**a**), *T*. *pyramidale* YN006 (**b**), *Trichoderma* sp. SRR12137155 (**c**), *T*. *velutinum* FJ002 (**d**), *T*. *pseudokoningii* TpsA (**e**), *T*. *citrinoviride* SRR18739368 (**f**), *T*. *ghanense* SC106 (**g**), *T*. *gracile* HK011 (**h**), *T*. *reesei* TreA (**i**), *T. longibrachiatum* XJ011 (**j**), *T. asperelloides* ZJ116 (**k**), and *T*. *asperellum* DQ-1 (**l**).

**Figure 4:**
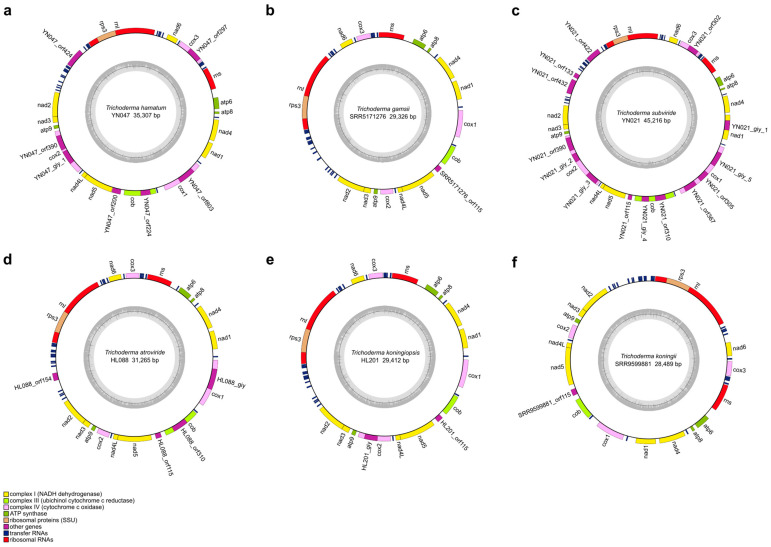
Mitogenome map of *T*. *hamatum* YN047 (**a**), *T*. *gamsii* SRR5171276 (**b**), *T*. *subviride* YN021 (**c**), *T*. *atroviride* HL088 (**d**), *T*. *koningiopsis* HL201 (**e**), and *T*. *koningii* SRR9599881 (**f**).

**Figure 5:**
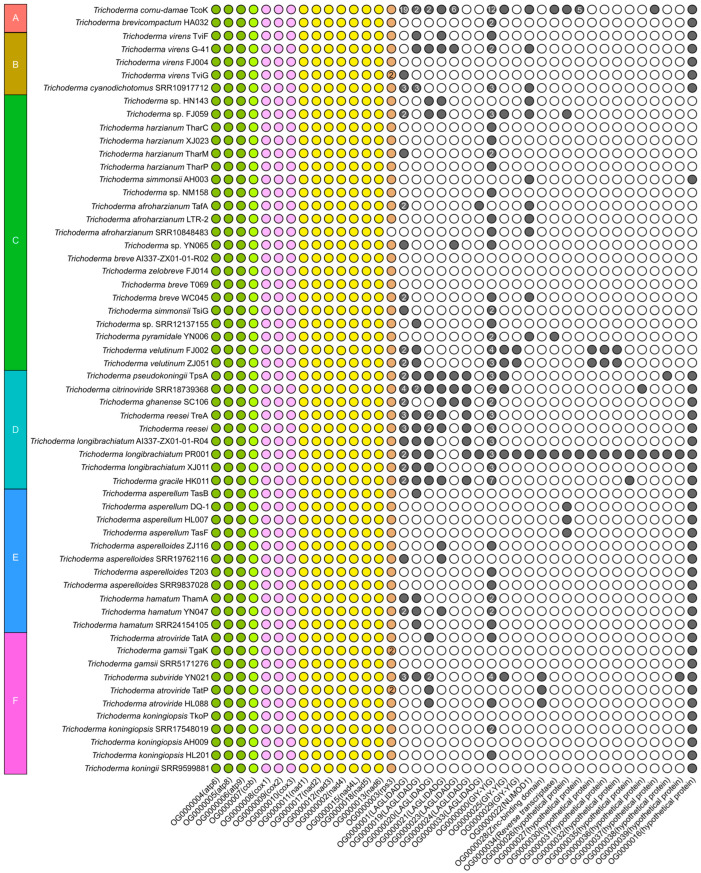
Comparative analysis of homologous gene families in the *Trichoderma* mitogenomes. The color-filled circle represents the presence of homologous genes, and the number represents the number of homologous genes. Blocks A–F were identified by phylogenetic relationships of *Trichoderma* species.

**Figure 6:**
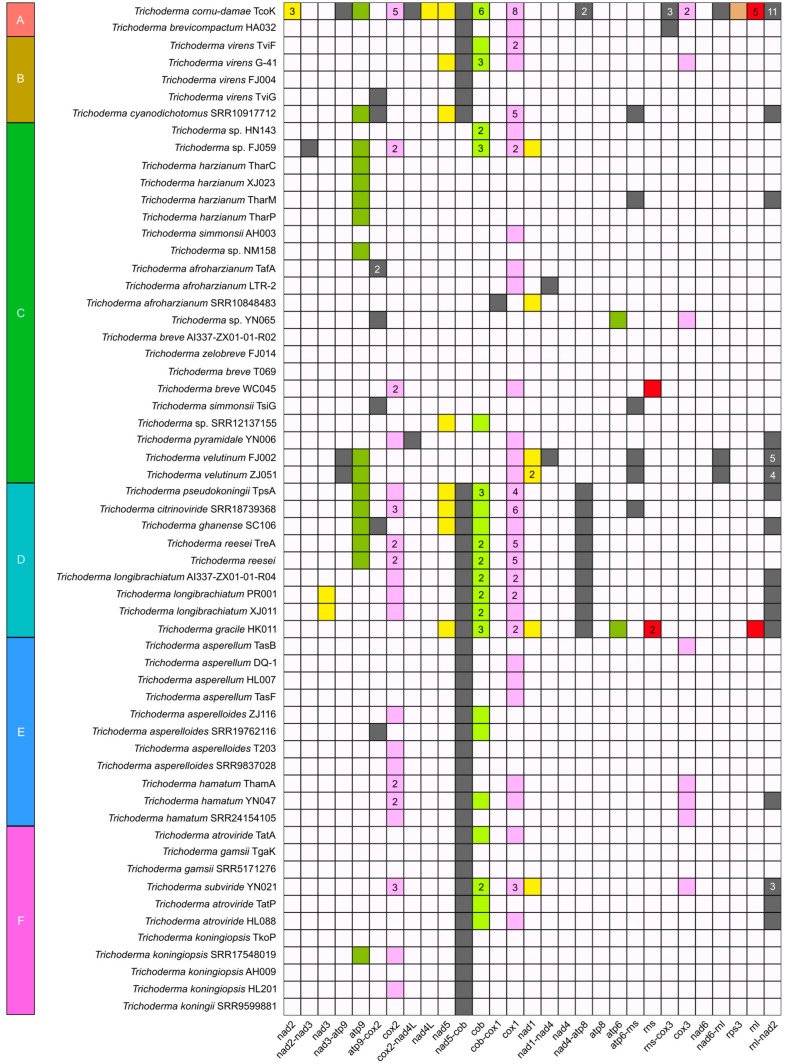
Comparative analysis of intron in the *Trichoderma* mitogenomes. The genes are presented in order of their position in the genome below the squares. The color-filled square represents the presence of intron in the gene or intergenic region, and the number represents the number of intron. Blocks A–F were identified by phylogenetic relationships of *Trichoderma* species.

In this study, we reported 42 *Trichoderma* mitogenomes, which were combined with 18 published *Trichoderma* mitogenomes from GenBank (accession numbers presented in Table 1), and 60 mitogenomes were annotated (Figure 2, Figure 3, Figure 4 and Figures S4–S6). Among the 42 newly reported *Trichoderma* mitogenomes, the complete mitogenomes of 13 *Trichoderma* species (*T. breve*, *T. longibrachiatum*, *T. velutinum*, *T. zelobreve*, *T. brevicompactum*, *T. gracile*, *T. ghanense*, *T. zeloharzianum*, *T. pyramidale*, *T. subviride*, *T. asperelloides*, *T. citrinoviride*, and *T. cyanodichotomus*) have been reported for the first time. The 60 *Trichoderma* mitogenomes were circular and exhibited considerable variations in genome size, ranging from 26,276 bp (*T. breve* AI337-ZX01-01-R02 and *T. zelobreve* FJ014) to 94,608 bp (*T. cornu-damae* KA19-0412C). The GC content of these *Trichoderma* mitogenomes ranged from 26.86% (*T. brevicompactum* HA032) to 28.29% (*T. koningii* SRR9599881). The annotation results revealed that the 60 *Trichoderma* mitogenomes encoded a set of 38–96 genes, including two rRNA genes (*rnl* and *rns*), 16–30 tRNA genes, and 15–68 protein-coding genes (PCGs) (Table 1). Each of these *Trichoderma* mitogenomes encodes 15 core PCGs, including three cytochrome c oxidase subunits (*cox1-3*), apocytochrome b (*cob*), three ATP synthase subunits (*atp6*, *atp8-9*), seven subunits of NADH dehydrogenase (*nad1-6*, *nad4L*), and ribosomal protein S3 (*rps3*). In addition, these *Trichoderma* mitogenomes encode GIY-YIG endonuclease, LAGLIDADG endonuclease, and hypothetical protein genes of unknown function. The number of GIY-YIG endonucleases ranged from 0 to 13, the number of LAGLIDADG endonucleases ranged from 0 to 30, and the number of hypothetical protein genes ranged from 0 to 11 (Figure 2, Figure 3, Figure 4 and Figure 5). Furthermore, these *Trichoderma* mitogenomes contained 0–53 introns, encoding *rps3*, GIY-YIG endonuclease, LAGLIDADG endonuclease, and hypothetical protein genes. Among them, the mitogenomes of some *Trichoderma* strains contained one or more introns in *atp9*, *cox2*, *cob*, *cox1* genes, the intergenic region between *nad5* and *cob*, and the intergenic region between *rnl* and *nad2*. Moreover, among the core protein-coding genes, the mitogenomes of some strains rarely contained introns compared to other genomes, including the *nad2*, *nad4*, and *rps3* genes of *T. cornu-damae* Tcok, the *nad3* genes of *T. longibrachiatum* PR001 and *T. longibrachiatum* XJ011, and the *atp6* gene of *Trichoderma* sp. YN065 and *T. gracile* HK011 (Figure 6). In a previous study, only one rRNA gene (*rns*) was reported in *T. asperellum* B05 (TasB), and the *atp9* gene was absent in *T. gamsii* KUC1747 (TgaK) [9]. However, our annotation identified the presence of the *rnl* gene of TasB and the *atp9* gene of TgaK (Figure S4). Furthermore, the *rps3* gene of *T. cornu-damae* KA19-0412C (TcoK) has not previously been reported to be located within *rnl* gene [26]. In contrast, our annotations indicated that the *rnl* gene of TcoK was significantly longer, and more coding genes were present in the intron besides the *rps3* gene (Figure 1, Supplementary Table S3). We found that among the 60 *Trichoderma* mitogenomes in this study, 56 exhibited a single nucleotide overlap between the stop codon of the *nad4L* gene and the start codon of the *nad5* gene, which was different from those in the four *T. asperellum* mitogenomes of TasB, TasF, DQ-1, and HL007. The length of the interval between *nad4L* and *nad5* in their mitogenomes was 456 bp, except for TasF, which was 454 bp long (Supplementary Table S3).

**Figure 7:**
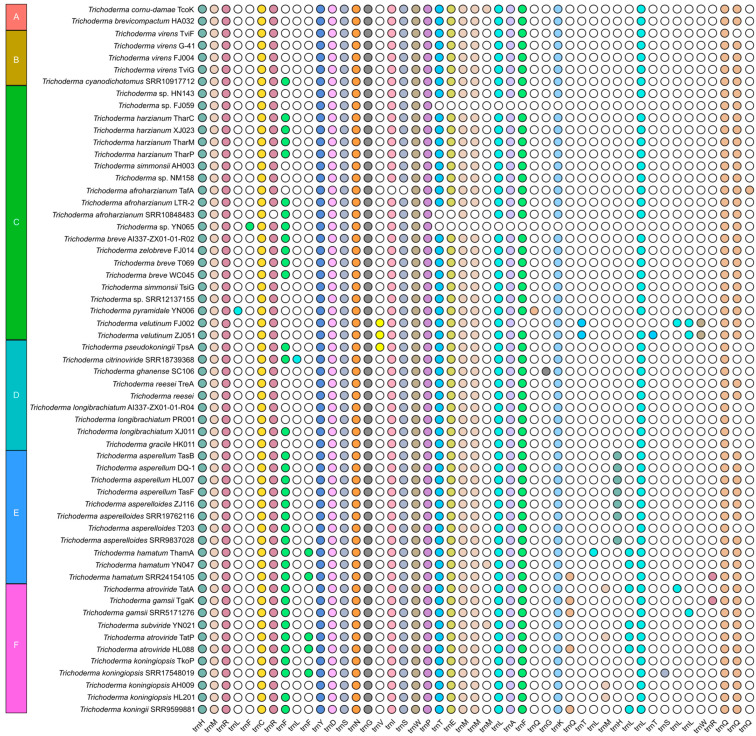
Comparative analysis of tRNA in the *Trichoderma* mitogenomes. The tRNAs are presented in order of their position in the genome below the circles. The color-filled circle represents the presence of the tRNA. Blocks A–F were identified by phylogenetic relationships of *Trichoderma* species.

For whole *Trichoderma* mitogenomes, the order of the tRNA genes in the genome is highly conserved; however, there are differences in the number and copy number of the tRNA genes. *Trichoderma* sp. FJ059 and *Trichoderma* sp. YN065 contained fewer tRNA genes, which lacked the *trnT*, *trnE*, *trnL*, *trnA*, and *trnK* genes. In addition, both strains contained only one *trnM* gene, whereas the other strains contained three *trnM* gene copies. Furthermore, *T. cornu-damae* Tock, *T. koningiopsis* AH009, and several other *Trichoderma* mitogenomes have been observed to possess four copies of the *trnM* gene. The mitogenome of *T. ghanense* SC106 contained two copies of the *trnG* gene, and the number of *trnW* copies was two in both *T. velutinum* FJ002 and *T. velutinum* ZJ051, whereas only one was predicted in the other strains. The mitogenomes of *T. hamatum* SRR24154105 and *T. gamsii* TgaK both had three copies of the *trnR* gene, whereas two *trnR* copies were present in all strains, with the exception of *T. afroharzianum* SRR10848483, which displayed a single copy of this gene. The mitogenome of *T. koningiopsis* SRR17548019 exhibited three copies of the *trnS* gene, with two copies of this gene being identified in all strains except *T. afroharzianum* TafA, which displayed only one copy of the *trnS* gene. Furthermore, most *Trichoderma* strains displayed multiple copies of *trnF* and *trnQ* in their mitogenomes (Figure 7).

### 2.3. Phylogenetic Analysis at Whole-Genome Level

**Figure 8:**
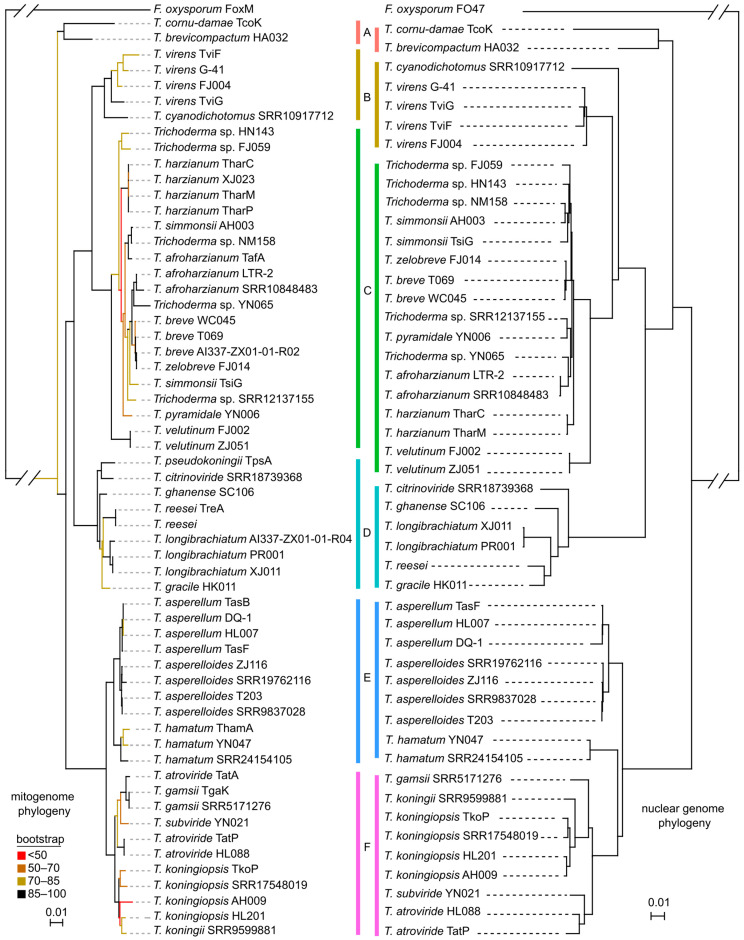
Phylogenetic tree based on the mitogenome (**left**) and nuclear genome (**right**) of *Trichoderma*. For the mitogenome phylogeny, it was inferred from the whole mitogenome sequences of 59 *Trichoderma* strains, based on Maximum likelihood (ML) methods. The best model of GTR + I + G with a bootstrap value of 1000 replicates was used to construct the phylogeny, and the *Fusarium oxysporum* mh2-2 (FoxM) was used as an outgroup. The 59 *Trichoderma* strains were clustered into six main evolutionary branches (A–F), which were represented by different color blocks. Regarding the nuclear genome phylogeny, it was constructed based on single-copy genes from 48 *Trichoderma* nuclear genomes, with *F. oxysporum* FO47 as the outgroup. The bootstrap values of the tree nodes were coded with different colors.

Phylogenetic tree inferred from the whole mitogenome sequences of 59 *Trichoderma* strains based on ML methods. The 59 strains of *Trichoderma* were classified into six major evolutionary branches (A–F) (Figure 8). Branch A contained *T. cornu-damae* and *T. brevicompactum*; branch B contained *T. virens* and *T. cyanodichotomus*; branch C contained *T. harzianum*, *T. afroharzianum*, *T. simmonsii*, *T. breve*, *T. zelobreve*, *T. pyramidale*, and *T. velutinum*; branch D contained *T. pseudokoningii*, *T. ghanense*, *T. reesei*, *T. longibrachiatum*, *T. citrinoviride*, and *T. gracile*; branch E contained *T. asperellum*, *T. hamatum*, and *T. asperelloides*; branch F contained *T. atroviride*, *T. subviride*, *T. gamsii*, *T. koningii*, and *T. koningiopsis*.

In the inferred phylogenetic tree, node support between the five branches (B–F) was high (91% or 100%), except for evolutionary branch A, which had low node support with the other branches (74%) (Figure S7). Most nodes within each branch exhibited high levels of support, with values ranging from 85% to 100%. However, some of the branch nodes within branch C exhibited 70–85% support, whereas individual branches demonstrated less than 50% support. Notably, this branch encompassed 21 *Trichoderma* strains, representing the highest species diversity (Figure 8). In addition, the phylogenetic tree constructed based on 2830 single-copy genes of 48 *Trichoderma* nuclear genomes had a topology similar to that of the tree based on the mitogenome (Figure 8, Figures S7 and S8), supporting the capability to explore the divergence of *Trichoderma* species based on mitogenomes.

### 2.4. Comparative Analysis of Trichoderma Mitogenomes from Different Evolutionary Branches

**Figure 9:**
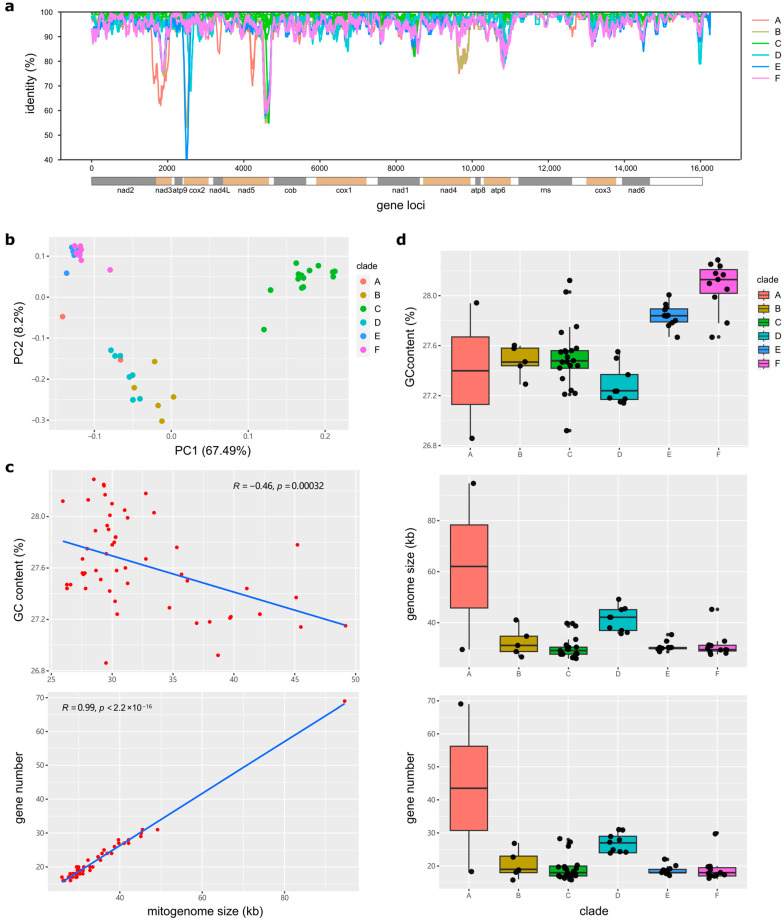
Comparison of characteristics between *Trichoderma* mitogenomes: (**a**) Consistent characterization of the *Trichoderma* mitogenomes. The identity value of the conserved region sequences of *Trichoderma* mitogenomes was calculated by sliding window analysis with a window length of 100 bp and a step size of 10 bp. The *T. breve* T069 sequence was used as the reference to identify the gene location information below the *x*-axis. (**b**) Principal component analysis (PCA) according to the conserved sequences of *Trichoderma* mitogenomes. (**c**) Relationship between GC content, coding gene number, and genome size for *Trichoderma* mitogenomes (excluding *T. cornu-damae* KA19-0412C in the relationship between GC content and genome size). (**d**) Comparative analysis of the GC content, gene size, and coding gene number of the *Trichoderma* mitogenomes of six evolutionary branches. Branches A–F and the representative colors in (**a**,**b**,**d**) are consistent with Figure 8.

Among the 58 *Trichoderma* mitogenomes (refer to 4.6, Materials and methods section), we identified 16,231 bp conserved sequences at the whole-mitogenome level, which represented highly conserved sequence identities, distinctly changed patterns, and mutational hot spots among mitogenomes from six distinct branches (Figure 9a). The results indicated that these mitogenomes exhibited a high level of conservation, with most identity values exceeding 90%. However, hotspots of variation were observed in *nad3*, *cox2*, and *nad5*. The principal regions of variation in branch A were observed in the *nad3*, *nad5*, and *nad4* gene regions, branch B in the *cox2*, *nad5*, and *nad4* gene regions, branch C in the *nad5* gene region, branch D in the *cox2* and *nad5* gene regions, branch E in the *cox2* and *nad5* gene regions, and branch F in the *nad3*, *nad5*, and *atp6* gene regions. These results revealed that the three PCGs among the *Trichoderma* mitogenomes could be considered potential molecular markers for phylogenetic analyses.

The principal component analysis supported the divergence of the six major branches of these mitogenomes (Figure 9b). Clustered mitogenomes from the six branches were clearly identified. The two strains from the early divergent branch A were located between strains from branches D and E. The strains from branches B, C, and D were separated. The strains from branches E and F were close to each other, which is consistent with their recent divergence in phylogeny.

To further explore the evolutionary features of the *Trichoderma* mitogenome, a comparative analysis was conducted of the GC content, genome size, number of coding genes, introns, and tRNAs of different evolutionary branches of the *Trichoderma* mitogenome. The GC content of the 60 *Trichoderma* mitogenomes ranged from 26.86% (*T. brevicompactum* HA032) to 28.29% (*T. koningii* SRR9599881), and that of the coding genes ranged from 15 (*T. zelobreve* FJ014, *T. breve* T069 and AI337-ZX01-01-R02) to 68 (*T. cornu-damae* Tcok) (Table 1). Correlation analysis revealed a negative correlation between GC content and *Trichoderma* mitogenome size and a positive correlation between coding gene number and *Trichoderma* mitogenome size (Figure 9c). The E (*T. asperellum*, *T. hamatum*, and *T. asperelloides*) and F (*T. atroviride*, *T. subviride*, *T. gamsii*, *T. koningii*, *and T. koningiopsis*) branches exhibited a higher GC content in the mitogenome. In contrast, the D (*T. pseudokoningii*, *T. ghanense*, *T. reesei*, *T. longibrachiatum*, *T. citrinoviride*, *and T. gracile*) branch displayed a larger genome size and a higher number of coding genes and introns (Figure 6 and Figure 9d).

Furthermore, *T. cornu-damae* Tcok from branch A had two introns in the intergenic region between the *nad4* and *atp8* genes, and the *Trichoderma* mitogenomes of branch D contained a single intron in the intergenic region between the *nad4* and *atp8* genes, which were different from the mitogenomes in other branches (Figure 6). *Trichoderma* mitogenomes in branch C were devoid of predicted introns in the *nad5* and *cob* intergenic regions. In contrast, the mitogenomes of the other five branches contained introns in the *nad5* and *cob* intergenic regions. The mitogenomes of certain strains belonging to branches E and F exhibited an increased number of copies of tRNA genes *trnF*, *trnM*, *trnQ*, *trnL*, and *trnH* (Figure 7).

### 2.5. Evolutionary Selection on PCGs of the Trichoderma Mitogenome

**Figure 10:**
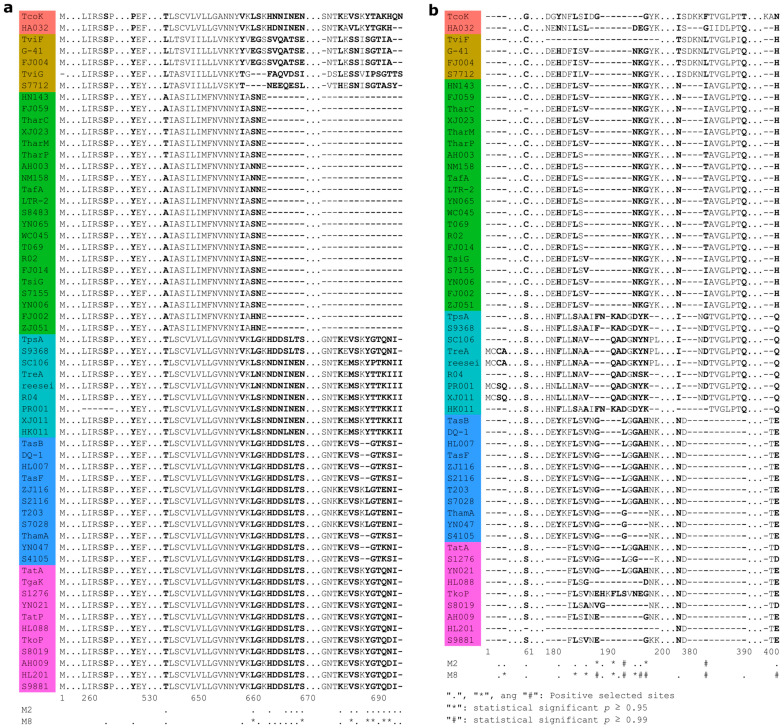
Positive selection sites across *nad5* (**a**) and *rps3* (**b**) in *Trichoderma* mitogenome. Positive selection sites were identified using Bayes Empirical Bayes dN/dS values and labeled with symbols of “.”, “*” (*p* ≥ 0.95) and “#” (*p* ≥ 0.99). The different colors represent the A–F branches as shown in Figure 8. TcoK: *T*. *cornu-damae* TcoK, HA032: *T*. *brevicompactum* HA032, TviF: *T*. *virens* TviF, G-41: *T*. *virens* G-41, TviG: *T*. *virens* TviG, FJ004: *T*. *virens* FJ004, S7712: *T. cyanodichotomus* SRR10917712, HN143: *Trichoderma* sp. HN143, FJ059: *Trichoderma* sp. FJ059, TharC: *T*. *harzianum* TharC, XJ023: *T. harzianum* XJ023, TharM: *T*. *harzianum* TharM, TharP: *T*. *harzianum* TharP, AH003: *T. simmonsii* AH003, NM158: *Trichoderma* sp. NM158, TafA: *T*. *afroharzianum* TafA, LTR-2: *T*. *afroharzianum* LTR-2, S8483: *T*. *afroharzianum* SRR10848483, YN065: *Trichoderma* sp. YN065, WC045: *T*. *breve* WC045, T069: *T*. *breve* T069, R02: *T*. *breve* AI337-ZX01-01-R02, FJ014: *T. zelobreve* FJ014, TsiG: *T. simmonsii* TsiG, S7155: *Trichoderma* sp. SRR12137155, YN006: *T*. *pyramidale* YN006, FJ002: *T*. *velutinum* FJ002, ZJ051: *T*. *velutinum* ZJ051, TpsA: *T*. *pseudokoningii* TpsA, S9368: *T*. *citrinoviride* SRR18739368, SC106: *T*. *ghanense* SC106, TreA: *T*. *reesei* TreA, reesei: *T*. *reesei* reesei, R04: *T. longibrachiatum* AI337-ZX01-01-R04, PR001: *T. longibrachiatum* PR001, XJ011: *T. longibrachiatum* XJ011, HK011: *T*. *gracile* HK011, TasB: *T*. *asperellum* TasB, DQ-1: *T*. *asperellum* DQ-1, HL007: *T*. *asperellum* HL007, TasF: *T*. *asperellum* TasF, ZJ116: *T. asperelloides* ZJ116, S2116: *T. asperelloides* SRR19762116, T203: *T. asperelloides* T203, S7028: *T. asperelloides* SRR9837028, ThamA: *T*. *hamatum* ThamA, YN047: *T*. *hamatum* YN047, S4105: *T*. *hamatum* SRR24154105, TatA: *T*. *atroviride* TatA, TgaK: *T*. *gamsii* TgaK, S1276: *T*. *gamsii* SRR5171276, YN021: *T*. *subviride* YN021, TatP: *T*. *atroviride* TatP, HL088: *T*. *atroviride* HL088, TkoP: *T*. *koningiopsis* TkoP, S8019: *T*. *koningiopsis* SRR17548019, AH009: *T*. *koningiopsis* AH009, HL201: *T*. *koningiopsis* HL201, S9881: *T*. *koningii* SRR9599881.

To determine whether the 15 core PCGs from *Trichoderma* mitogenomes are evolving under positive selection pressure or not, we calculated nonsynonymous/synonymous substitution rate ratios (dN/dS) for these genes using the CODEML program in PAML with site models M1 (neutral), M2 (selection), M7 (beta), and M8 (beta & ω). For the *nad5* gene, the LRT statistic for comparing M7 [lnL(log likelihood value) = −7686.569999] and M8 (lnL = −7678.380104) is 16.37979 [2Δ = 2 × (7686.569999 − 7678.380104) = 16.37979], with a *p*-value = 2.774 × 10^−4^ using the Chi-square test (with df = 2). For the *rps3* gene, we calculated the value of the LRT statistic between M7 and M8 (2Δ = 45.262836, *p*-value = 1.484 × 10^−10^, the Chi-square test with df = 2), as well as M1 and M2 (2Δ = 32.447006, *p*-value = 9 × 10^−8^) (Table 2, Supplementary Table S4). Moreover, we explored 18 sites in *rps3* with values of dN/dS > 1, 11 sites were considered statistically significant (*p* ≥ 0.95), and 23 sites in *nad5* with values of dN/dS > 1, and seven sites were considered statistically significant (*p* ≥ 0.95) (Figure 10). The results of the log-likelihood test indicated that *atp9*, *cob*, and *nad4L* genes were positively selected in *Trichoderma* mitogenome with a chi-squared test *p*-value < 0.05 (Supplementary Table S4).

## 3. Discussion

In the present study, we annotated and analyzed 60 *Trichoderma* mitogenomes. The mitogenomes of *T. breve* AI337-ZX01-01-R02 and *T. zelobreve* FJ014 (26,276 bp) were the smallest *Trichoderma* mitogenomes, followed by *T. koningiopsis* POS7 (TkoP) (27,560 bp) [27]. These *Trichoderma* mitogenomes generally included 15 core protein-encoding genes (*cox1-3*, *cob*, *atp6*, *atp8-9*, *nad1-6*, *nad4L*, and *rps3*) and two conserved ribosomal RNA genes (*rnl* and *rns*), which is consistent with previous reports on *Trichoderma* mitogenomes [11,27,28]. Furthermore, the order of the 15 core PCGs, two rRNA genes, and some tRNA genes in *Trichoderma* mitogenomes was highly conserved. A single nucleotide overlap between the stop codon of the *nad4L* gene and the start codon of the *nad5* gene in 56 *Trichoderma* mitogenomes, consistent with the characteristics of *Trichoderma* mitogenomes reported previously [28]. Interestingly, all four strains that did not exhibit this common phenomenon in their mitogenomes were *T. asperellum*. The reason for this particularity of the species *T. asperellum* is worth further investigation.

This study newly reported 42 *Trichoderma* mitogenomes, and the mitogenome phylogeny revealed the phylogenetic placement of at least 30 species, supported by nuclear phylogeny at the whole-genome level (Figure 8). Ongoing advances in genomics and biotechnology have facilitated the sequencing and assembly of mitogenomes. As shown in this study, assemblers [29,30,31] can obtain complete mitogenomes based on the assembly of short or long sequence reads. Mitogenomic phylogeny offers the opportunity to identify *Trichoderma* spp. The molecular identification of *Trichoderma* spp. based on DNA barcodes with little genetic information remains challenging. A survey on the identification of two *Trichoderma* isolates based on the ITS, *tef1*, and *rpb2* genes showed that 21% (10/47) of the experts correctly identified both strains [4]. Compared with the minimal size of 31 Mb for nuclear genomes [7,32,33,34,35,36], the sizes of almost all mitogenomes are less than 50 kb, with a maximal size of 94 kb [9,11,16,26,27,28,34,37,38,39,40], suggesting the easy and efficient use of mitogenomes for correct species identification.

Although fungal mitogenomes exhibit considerable variability in terms of size, structure, and gene order [20], the gene order of Hypocreales mitogenomes is conserved [41], suggesting that it is possible to infer phylogenetic relationships among *Trichoderma* species using one *Fusarium* strain as an outgroup. Researchers have frequently used the amino acid sequences of mitogenome core protein-encoding genes for phylogenetic analysis, resolving numerous ambiguous phylogenetic issues [42]. This approach has become the standard methodology for phylogenetic analyses [43]. In addition, studies have used mitochondrial intergenic region sequences to analyze phylogeny and identify molecular phylogenies within and between fungal species [44,45]. The core protein-coding genes, rRNA genes, and the majority of the tRNA gene sequences of the 60 *Trichoderma* mitogenomes analyzed in this study exhibited high levels of conservation (Supplementary Table S3). Considering the aforementioned findings, we used the mitochondrial whole-genome sequence for phylogenetic analysis, resulting in a phylogenetic tree that was largely concordant with the classification information for these *Trichoderma* species (Figure 8). Furthermore, the evolutionary branches of *Trichoderma* obtained by our research exhibited notable similarity to those reported in previous studies [4,7]. The detailed comparison of tree topologies between mitogenome and nuclear genome lineages showed little difference, such as the phylogenetic placement of branch A, which may be due to random sorting of ancestral lineages during the short internode, homoplasy in the mtDNA data, or both [46]. However, distinct branches were observed in the mitogenome and nuclear genome phylogenies (Figure 8). The phylogenetic analysis methods and results presented in this study serve as valuable references for the classification and identification of *Trichoderma*. In addition, this study identified three mutation hotspot regions in the *nad3*, *cox2*, and *nad5* genes of the *Trichoderma* mitogenome. These three PCGs (*nad3*, *cox2*, and *nad5*) have the potential to serve as molecular markers for phylogenetic analyses, thereby clarifying taxonomic ambiguities associated with *Trichoderma* spp. The phylogenetic tree constructed from the concatenated sequences of *nad3*, *cox2*, and *nad5* genes of 59 *Trichoderma* strains exhibits a topology similar to that of the mitogenome phylogeny (Figure 8 and Figure S9), which represents the ability to recognize strains from different branches.

The GC content of the *Trichoderma* mitogenomes analyzed in this study ranges from 26.86% to 28.29%, with 0–53 introns and 16–30 tRNA genes. Variations in the size of the *Trichoderma* mitogenome were primarily associated with the number and length of introns and accessory genes (Table 1, Figure 9, Supplementary Table S3). *Trichoderma cornu-damae* TcoK (94,608 bp) had the largest mitogenome and contained 53 introns, whereas the remaining mitogenomes ranged in size from 26,276 bp to 49,170 bp and contained introns ranging from 0 to 15, supporting the previous discovery of intronic regions as major size contributors in fungal mitogenomes [41]. The smallest mitogenomes of *T. breve* AI337-ZX01-01-R02, *T. zelobreve* FJ014, and another smaller *Trichoderma* mitogenome (*T. breve* T069) had no introns (Figure 2, Figure 3 and Figure S5). Furthermore, comparative analysis revealed that the genome size, number of genes, GC content, tRNA copy number, and number of introns in the mitogenomes of different *Trichoderma* branches exhibited notable variation. The mitogenomes of the E and F branches (*T. asperellum*, *T. hamatum*, and *T. atroviride*, etc.) of various biocontrol *Trichoderma* species exhibited high GC content, and some strains displayed multiple copies of tRNA within their mitogenomes. The mitogenome size of the D branch of the industrial fungus, *T. reesei* is larger, with a greater number of coding genes and introns. Except for *T. cornu-damae* Tcok, which had two introns in the *nad4* and *atp8* intergenic regions, only *Trichoderma* mitogenomes in the D branch contained one intron in the *nad4* and *atp8* intergenic regions (Figure 6, Figure 7 and Figure 9d). The genomic GC content of an organism is influenced by mutation bias, selection, and biased recombination associated with DNA repair. This can be used as an indicator reflecting evolutionary processes [47,48]. The high rate of evolution of the mitogenome allows the number and positioning of its introns to explain the different variabilities within different strains within the same genus or even within the same species [49]. Furthermore, according to previous reports [50], both conserved mitochondrial genes and highly variable regions in fungal mitogenomes are involved in host phenotypic plasticity. Therefore, the significant differences in the mitogenomes of biocontrol *Trichoderma* and industrial *Trichoderma* from different evolutionary branches revealed in this study provide important information for explaining the correlation between their differences in parasitic and biocontrol abilities, as well as for studying the adaptive evolution of *Trichoderma*.

Gene selection pressure analysis is an important method for understanding species adaptability, revealing the evolutionary paths of genes, and studying their inheritance and evolution [51]. It provides a perspective and method for gaining an in-depth understanding of biological adaptability. An analysis of coding gene sequences in human mitogenomes revealed that natural selection shaped regional mtDNA variations [52]. Variability has been found in the evolutionary rates of genes encoded in animal mitogenomes, which are influenced by parasitic lifestyle and locomotory capacity [53]. Our selection pressure analysis revealed that *atp9*, *cob*, *nad4L*, *nad5*, and *rps3* were positively selected during evolution. There were multiple positive selection sites in the amino acid sequences of *nad5* and *rps3*, with the observed changes at these sites exhibiting a high degree of correlation with the evolutionary branches of the phylogeny (Figure 10). Mutations in sequences from these positively selected sites in the mitogenome may be affected by lifestyle changes in *Trichoderma* spp. Ecophysiological and lifestyle changes may also have resulted in species diversification [1]. For species in branch C of the phylogenetic tree (Figure 8), divergent events may have occurred recently, and they possessed special mutations (Figure 10). Similar patterns were observed in species from branches D, E, and F (Figure 8 and Figure 10). Mutations in *nad5* render it an ideal molecular marker for systematic evolution and taxonomic identification. It has been successfully used to study phylogeny and genetic variation in numerous organisms [54,55,56,57]. Sequence changes in genes such as the ancient gene *rps3* may reflect the evolution of the fungal mitogenome [58]. Future studies on the evolutionary changes in genes and phenotypic traits may provide a comprehensive understanding of the evolution of positively selected genes in *Trichoderma*.

## 4. Materials and Methods

### 4.1. Molecular Identification of 60 Trichoderma Strains

Sixty *Trichoderma* strains were selected for mitogenome analysis. To correctly identify the species names of these strains, we performed molecular identification analysis based on three genes: ITS, *tef1*, and *rpb2*. An alignment of the ITS, *tef1*, and *rpb2* sequences was performed using MUSCLE (version 3.8.31) [59]. The aligned genes were concatenated using the Perl script (global_alignment_single_copy_genes.pl; https://github.com/linrm2010/global_alignment_single_copy_genes/; accessed on 7 November 2022). Poorly aligned regions were removed using Gblocks (version 0.91b) [60]. Raxml-ng (version 1.1.0) [61] with a bootstrap value of 500 and MEGA (version X) [62] with a bootstrap value of 1000 were used to construct the phylogenetic tree [63] under the best model of GTR + I + G, which was identified using jModelTest (version 2.1.4) [64].

### 4.2. Assembly of the Trichoderma Mitogenomes

The total genomic DNA of each of the 29 strains was isolated from freeze-dried mycelia. DNA libraries were constructed, and the sequenced data were produced using the Illumina Xten platform at Biomarker Company (Beijing, China). PacBio HiFi sequencing data were produced for the T069 strain and nanopore sequencing data were produced for the HL201 strain. In addition, the reported Illumina short reads of 11 strains and BGISEQ-500 short reads of two strains were downloaded from NCBI. Based on these sequenced data of 42 strains, we used SPAdes (version 3.15.4) [29] for genome assembly, which yielded 40 mitogenomes, and used Hifiasm (version 0.16.1) [30] for genome assembly of the T069 strain, as well as used Canu (version 1.8) [31] for genome assembly of HL201 strain. During the assembly process, the mitochondrial DNA contigs were identified by aligning assembly sequences against previously reported *Trichoderma* mitogenomes, which showed high sequence similarity. For each strain, we may always obtain one mitochondrial contig containing overlapping sequences at the beginning and end of the contig. We manually improved the contig by removing overlapping sequences at the end. Finally, we obtained 42 mitogenomes, including 38 complete mitogenomes and four mitogenomes with gaps (Supplementary Table S1).

### 4.3. Trichoderma Mitogenome Annotation and Visualization

We annotated 42 *Trichoderma* mitogenomes reported in this study and 18 reported mitogenomes of *Trichoderma* from NCBI (Table 1). We uploaded the mitogenome sequence to MITOS (https://usegalaxy.org/root?tool_id=toolshed.g2.bx.psu.edu%2Frepos%2Fiuc%2Fmitos2%2Fmitos2%2F2.1.3%20galaxy0 (accessed on 2 January 2024)) [65], with the parameter “Reference: RefSeq 63 fungi; Genetic Code: 4 Mold” and MFannot webserver (https://megasun.bch.umontreal.ca/apps/mfannot/ (accessed on 2 January 2024)) [66] with the parameter “Genetic Code: 4 Mold” for basic gene prediction. For protein-coding gene structure prediction, we used IGV [67] to uncover the varied prediction results between MITOS and MFannot and aligned the predicted amino acids against the downloaded mitochondrial sequences from the NCBI refseq database (https://ftp.ncbi.nlm.nih.gov/refseq/release/mitochondrion/ (accessed on 2 January 2024)) using Basic Local Alignment Search Tool for Proteins (BLASTP) [68]. In addition, we submitted the amino acid sequence of the predicted protein-coding gene to NCBI CDD (https://www.ncbi.nlm.nih.gov/cdd (accessed on 2 January 2024)) [69] for domain annotation. For the annotation of non-coding RNAs, we used tRNAscan-SE (version 2.0, http://trna.ucsc.edu/tRNAscan-SE/ (accessed on 2 January 2024)) [70] with the parameters of “sequence source: other mitochondrial; Search mode: default; Genetic Code for tRNA Isotype Prediction: Mold & Protozoan Mito” to predict transfer RNAs (tRNAs), combined with the tRNA prediction results of MITOS and MFannot, the set of prediction results of the three methods was obtained as the annotation results of tRNA. Ribosomal RNAs (rRNAs) were identified based on the prediction results of MITOS, MFannot, Rfam (https://rfam.org/ (accessed on 2 January 2024)) [71], RNAweasel (https://megasun.bch.umontreal.ca/apps/rnaweasel/ (accessed on 2 January 2024)) [72], as well as the rRNA reference sequences collected by the authors.

For repeat sequence annotation, we used the Tandem Repeats Finder (https://tandem.bu.edu/trf/trf.html (accessed on 2 January 2024)) [73] to predict the tandem repeat sequences. We used SFMA (https://github.com/linrm2010/SFMA; accessed on 30 Juanary 2024) to mask the predicted gene sequence and submitted the masked sequence to DNA Analyzer Palindrome (http://palindromes.ibp.cz/#/en/palindrome (accessed on 2 January 2024)) [74] with the parameters of “Size: 6–30; Spacer: 0–10; Mismatches: 0” to identify the reverse repeat sequence.

We combined the above analysis results using SFMA to obtain the tbl file, then table2asn (https://ftp.ncbi.nlm.nih.gov/asn1-converters/by_program/table2asn/ (accessed on 2 January 2024)) was used to generate the GenBank file, which was uploaded to OGDRAW (https://chlorobox.mpimp-golm.mpg.de/OGDraw.html (accessed on 2 January 2024)) [75] to generate the mitogenome map.

The detailed operational procedures and scripts used can be viewed in the SFMA. Detailed information on the annotations of the 60 *Trichoderma* mitogenomes is provided in Supplementary Table S3, and nucleotide sequence data reported for 13 mitogenomes are available in the Third Party Annotation Section of the DDBJ/ENA/GenBank databases under the accession numbers TPA: BK068259-BK068263, BK068321-BK068328 (Table 1).

### 4.4. Identification of Orthologous Gene Clusters

Based on the annotated amino acid sequences of genes from 60 *Trichoderma* mitogenomes and *Fusarium oxysporum* Mh2-2 mitogenome [76] that was selected as an outgroup for phylogenetic analysis, we used OrthoFinder (version 2.5.4) [77] to identify the orthologous groups of these genes. The originally analyzed results showed that the *nad2* and *nad5* genes from some *Trichoderma* mitogenomes were classified into one group, which was manually improved. A total of 39 groups were identified, including 15 groups of single-copy genes (*atp6*, *atp8-9*, *cob*, *cox1-3*, *nad1-6*, *nad4L*, and *rps3*), three groups of GIY-YIG endonucleases, and seven groups of LAGLIDADG endonucleases (Supplementary Table S2).

### 4.5. Phylogenetic Analysis

The whole-genome sequences of 59 *Trichoderma* mitogenomes were used to reconstruct phylogenetic relationships. The previously reported *T. harzianum* TharA was not included in the sliding window analysis of sequence identities among the 60 mitogenomes, suggesting a significant difference between TharA and other mitogenomes. *Trichoderma* mitogenomes are circular, and the order of the 15 core protein-coding genes (PCGs; *atp6*, *atp8-9*, *cob*, *cox1-3*, *nad1-6*, *nad4L*, and *rps3*), 2 rRNA genes (*rns* and *rnl*), and some tRNA genes in the genome is highly conserved (Supplementary Table S3). For each genome, the linear genome sequence was obtained with the *nad2* gene as the starting position of the whole genome sequence, which was submitted to the MAFFT webserver [78] for whole-genome sequence alignment. We used Gblocks (version 0.91b) [60] to remove low-quality aligned regions. The best model of GTR + I + G was identified using jModelTest (version 2.1.10) [64] to construct the phylogenetic tree with the parameter “-t ML -f -i -g 4 -AIC -BIC -a”. We used IQ-TREE (version 1.6.12) [79] to construct the maximum likelihood (ML) phylogenetic tree for the 59 mitogenomes with the parameter “-m GTR+I+G -b 1000”, setting *Fusarium oxysporum* mh2-2 as the outgroup. The ML phylogenetic tree was visualized using Archaeopteryx (version 0.9928) [80].

Of the 60 *Trichoderma* strains analyzed in this study, we obtained whole-genome sequences of nuclear genes from 48 strains (unpublished data). Based on the annotated amino acid sequences of nuclear genes from 48 *Trichoderma* genomes and *F. oxysporum* FO47 [81] that was selected as outgroup for phylogenetic analysis, we used OrthoFinder (version 2.5.4) [77] to identify 2830 single-copy genes and concatenated their alignments using the Perl script global_alignment_single_copy_genes.pl (https://github.com/linrm2010/global_alignment_single_copy_genes/; accessed on 7 November 2022). Poorly aligned regions were removed using Gblocks (version 0.91b) [60]. Raxml-ng (version 1.1.0) [61] with a bootstrap value of 500 was used to construct a phylogenetic tree under the best model of JTT+I+G+F, which was identified using ProtTest (version 3.4) [82].

### 4.6. Comparative Mitogenomic Analysis Across Trichoderma Species

The in-house Python script [83] was used to calculate the identity value of the conserved region sequences of *Trichoderma* mitogenomes using the sliding window approach and to visualize the results, excluding *T. harzianum* TharA (the reason discussed above) and *T*. *afroharzianum* SRR10848483. SRR10848483 showed extremely high variation at some sites owing to incompletely assembled mitogenome sequences, which may have led to incorrect results. Whole-genome alignment sequences were obtained using Gblocks and the *T. breve* T069 sequence was used as a reference. The window length was set to 100 bp with a step size of 10 bp. Identity values from the conserved sequence alignment results were used for principal component analysis (PCA) implemented in R (version 4.3.2).

### 4.7. Identification of Positively Selected Genes

For each core PCG, we used ClustalW (version 2.1) [84] to perform sequence alignment (excluding *T. harzianum* TharA, the reason as shown above; excluding *T. afroharzianum* SRR10848483, as an incomplete mitogenome with loss of the region encoding *rps3*; and excluding *T. atroviride* TatP, *T. gamsii* TgaK, and *T. virens* TviG, as the early appearance of the stop codon in *rps3* resulted in the prediction of two short Open Reading Frames lacking the complete sequence of *rps3*; Figure S6, Supplementary Table S3). The positive selection pressure on 15 core PCGs was detected using the CODEML program in PAML (version 4.10.7) [85] with site models Model1 (neutral), Model2 (selection), Model7 (beta), and Model8 (beta & ω). The likelihood ratio test (LRT) was performed to compare the likelihood differences between Model1 and Model2, and Model7 and Model8. Genes with a *p*-value < 0.05 using the Chi-square test (with df = 2; Model1 vs. Model2, and Model7 vs. Model8) were considered to be evolving under positive selection in *Trichoderma* (Supplementary Table S4).

## 5. Conclusions

In the present study, 42 *Trichoderma* mitogenomes were newly reported. In total, 60 *Trichoderma* mitogenomes were annotated by combining 18 previously published genomes with the new dataset. This study elucidated the structural characteristics of *Trichoderma* mitogenomes and their diversity among different *Trichoderma* species. The phylogenetic tree constructed based on the entire mitogenome provided insights into the phylogenetic relationships among *Trichoderma* species. Comparative analysis revealed significant differences in the mitogenome size, gene number, GC content, tRNA copy number, and intron number of *Trichoderma* in different branches. Furthermore, we identified three PCGs (*nad3*, *cox2*, and *nad5*) as mutation hotspots in the *Trichoderma* mitogenome. These genes can be used as potential molecular markers for further phylogenetic analyses. The results of the selection pressure analysis demonstrated that *atp9*, *cob*, *nad4L*, *nad5*, and *rps3* were subjected to positive selection during the evolutionary process. Our findings are beneficial for elucidating the phylogeny and evolutionary relationships of *Trichoderma* species, as well as for the development of potential molecular markers for phylogenetic analysis.

## Figures and Tables

**Table 1:** General features of the *Trichoderma* mitogenomes.

Species	Abbreviation	Accession	Size (bp)	GC (%)	PCGs	rRNAs	tRNAs
*T*. *afroharzianum*	TafA	ON764439.1 *	29,511	27.71	19	2	24
*T. afroharzianum* LTR-2	LTR-2	BK068323	29,060	27.51	17	2	26
*T. afroharzianum* SRR10848483	SRR10848483	BK068259	25,943	28.12	16	2	22
*T*. *asperelloides* ICC 012	SRR9837028	BK068324	28,621	27.89	17	2	27
*T*. *asperelloides* PK1J2	SRR19762116	BK068326	29,810	28.01	18	2	27
*T. asperelloides* T203	T203	BK068261	29,583	27.93	17	2	25
*T. asperelloides* ZJ116	ZJ116	PP952381	29,700	27.9	18	2	27
*T*. *asperellum* B05	TasB	NC_037075.1 *	29,999	27.78	17	2	27
*T*. *asperellum* DQ-1	DQ-1	PP933709	30,276	27.84	17	2	27
*T*. *asperellum* FT101	TasF	CP084950.1 *	30,285	27.84	17	2	27
*T. asperellum* HL007	HL007	PP952395	30,276	27.84	17	2	27
*T*. *atroviride* ATCC 26799	TatA	MN125601.1 *	32,758	28.18	18	2	29
*T. atroviride* HL088	HL088	PP952396	31,265	27.99	19	2	29
*T*. *atroviride* P1	TatP	CP084942.1 *	29,981	28.1	19	2	29
*T. breve* AI337-ZX01-01-R02	AI337-ZX01-01-R02	PP952387	26,276	27.47	15	2	26
*T*. *breve* T069	T069	PP933710	26,285	27.44	15	2	26
*T. breve* WC045	WC045	PP952403	31,266	27.48	19	2	26
*T. brevicompactum* HA032	HA032	PP952393	29,486	26.86	18	2	25
*T. citrinoviride* SRR18739368	SRR18739368	BK068321	49,170	27.15	30	2	25
*T*. *cornu-damae* KA19-0412C	TcoK	MW525445.1 *	94,608	27.94	68	2	26
*T. cyanodichotomus* SRR10917712	SRR10917712	BK068260	41,059	27.44	26	2	26
*T*. *gamsii* A5MH	SRR5171276	BK068328	29,326	28.24	16	2	29
*T*. *gamsii* KUC1747	TgaK	NC_030218.1 *	29,303	28.25	17	2	28
*T. ghanense* SC106	SC106	PP952402	38,007	27.18	23	2	25
*T. gracile* HK011	HK011	PP952394	45,495	27.14	30	2	24
*T*. *hamatum*	ThamA	MF287973.1 *	32,763	27.67	20	2	29
*T*. *hamatum* SRR24154105	SRR24154105	BK068327	30,164	27.8	18	2	28
*T. hamatum* YN047	YN047	PP952383	35,307	27.76	22	2	28
*T*. *harzianum* CBS 226.95	TharC	MN564945.1 *	27,632	27.55	16	2	26
*T*. *harzianum* MUT3171	TharM	NC_052832.1 *	29,791	27.42	18	2	26
*T*. *harzianum* PAR3	TharP	MZ713368.1 *	27,631	27.55	16	2	26
*T*. *harzianum*	TharA	MT263519.1 *	32,277	27.74	21	2	28
*T. harzianum* XJ023	XJ023	PP952405	27,757	27.56	16	2	26
*T*. *koningii* SRR9599881	SRR9599881	BK068263	28,389	28.29	16	2	28
*T. koningiopsis* SRR17548019	SRR17548019	BK068322	31,023	28.05	18	2	28
*T*. *koningiopsis* AH009	AH009	PP952386	28,026	28.13	16	2	26
*T. koningiopsis* HL201	HL201	PP952397	29,412	28.17	17	2	27
*T*. *koningiopsis* POS7	TkoP	MT816499.1 *	27,560	27.67	16	2	26
*T. longibrachiatum* AI337-ZX01-01-R04	AI337-ZX01-01-R04	PP952388	36,935	27.17	23	2	24
*T. longibrachiatum* PR001	PR001	PP952400	36,172	27.5	24	2	24
*T. longibrachiatum* XJ011	XJ011	PP952404	35,694	27.55	23	2	25
*T*. *pseudokoningii*	TpsA	OW971927.1 *	45,112	27.37	28	2	26
*T. pyramidale* YN006	YN006	PP952406	33,434	28.03	19	2	27
*T*. *reesei*	TreA	NC_003388.1 *	42,130	27.24	27	2	25
*T. reesei*	reesei	PP952401	42,130	27.24	27	2	25
*T. simmonsii* AH003	AH003	PP952385	27,813	27.44	16	2	25
*T*. *simmonsii* GH-Sj1	TsiG	MZ292901.1 *	28,668	27.58	17	2	25
*T. subviride* YN021	YN021	PP952382	45,216	27.78	29	2	28
*T. velutinum* FJ002	FJ002	PP952389	39,673	27.21	27	2	28
*T. velutinum* ZJ051	ZJ051	PP952380	39,750	27.22	26	2	30
*T. virens* FJ004	FJ004	PP952390	26,580	27.47	16	2	25
*T*. *virens* FT-333	TviF	CP071122.1 *	31,081	27.6	19	2	24
*T*. *virens* G-41	SRR9836993	BK068262	34,601	27.29	23	2	25
*T*. *virens* Gv29-8	TviG	CP071114.1 *	27,943	27.75	18	2	25
*T. zelobreve* FJ014	FJ014	PP952391	26,276	27.47	15	2	26
*Trichoderma* sp. FJ059	FJ059	PP952392	38,693	26.92	25	2	16
*Trichoderma* sp. HN143	HN143	PP952398	30,234	27.34	18	2	25
*Trichoderma* sp. M10	SRR12137155	BK068325	30,361	27.58	17	2	25
*Trichoderma* sp. NM158	NM158	PP952399	27,550	27.56	16	2	25
*Trichoderma* sp. YN065	YN065	PP952384	30,407	27.24	18	2	18

* The previously published *Trichoderma* mitogenomes are highlighted with asterisks.

**Table 2:** Log-likelihood values and parameter estimates for *nad5* and *rps3* genes in *Trichoderma* mitogenomes.

Gene	Model	ℓ	dN/dS	Estimates of Parameters
*nad5*	M1 (Nearly Neutral)	−7693.445639	0.0857	*p*_0_ = 0.92632 (*p*_1_ = 0.07368) *ω*_0_ = 0.01295
	M2 (Positive Selection)	−7693.445639	0.0857	*p*_0_ = 0.92632, *p*_1_ = 0.03912 *(p*_2_ = 0.03456) ω_0_ = 0.01295
	M7 (beta)	−7686.569999	0.1102	*p* = 0.02698, *q* = 0.21684
	M8 (beta & ω > 1)	−7678.380104	0.0853	*p*_0_ = 0.94591 (*p*_1_ = 0.05409) *p* = 0.07077, *q* = 2.62016, *ω*_s_ = 1.21899
*rps3*	M1(Nearly Neutral)	−4251.44464	0.1166	*p*_0_ = 0.90017 (*p*_1_ = 0.09983) *ω*_0_ = 0.01868
	M2(Positive Selection)	−4235.221137	0.2012	*p*_0_ = 0.89972, *p*_1_ = 0.07594 (*p*_2_ = 0.02434) *ω*_0_ = 0.02137
	M7(beta)	−4256.383312	0.1153	*p* = 0.03581, *q* = 0.27625
	M8(beta & ω > 1)	−4233.751894	0.1757	*p*_0_ = 0.96277 (*p*_1_ = 0.03723) *p* = 0.09916, *q* = 1.40487, *ω*_s_ = 3.16366

## Data Availability

Data are contained within the article and Supplementary Table S5.

## Supplementary Materials

The following supporting information can be downloaded at: https://www.mdpi.com/article/10.3390/ijms252212140/s1.
