# Supplementary material for: Assembly, Annotation, and Comparative Analysis of Mitochondrial Genomes in Trichoderma
Source: Int J Mol Sci. 2024 Nov 12;25(22):12140. doi: 10.3390/ijms252212140 (PMC11594488; doi:10.3390/ijms252212140)
Supplement: Supplementary file 1 [file ijms-25-12140-s001.zip › Supplementary Figure.pdf]

Score	Expect	Identities	Gaps	Strand
1962 bits(1062)	0.0	1064/1065(99%)	0/1065(0%)	Plus/Plus

```
T069        1     CTGTTCCGTGGTATCATGCGAAGGATGAACACTGAGTTGGCCAACTACCTGAGACGATGC  60
                  ||||||||||||||||||||||||||||||||||||||||||||||||||||||||||||
HMAS248844  22    CTGTTCCGTGGTATCATGCGAAGGATGAACACTGAGTTGGCCAACTACCTGAGACGATGC  81

T069        61    GTTGAGGGCAACCGACACTTCAACCTTGCTGTTGGTATCAAGCCCGGCACGCTTTCAAAC  120
                  ||||||||||||||||||||||||||||||||||||||||||||||||||||||||||||
HMAS248844  82    GTTGAGGGCAACCGACACTTCAACCTTGCTGTTGGTATCAAGCCCGGCACGCTTTCAAAC  141

T069        121   GGATTGAAGTATTCGCTTGCCACTGGCAACTGGGGTGATCAGAAGAAGGCCATGAGCTCA  180
                  ||||||||||||||||||||||||||||||||||||||||||||||||||||||||||||
HMAS248844  142   GGATTGAAGTATTCGCTTGCCACTGGCAACTGGGGTGATCAGAAGAAGGCCATGAGCTCA  201

T069        181   ACTGCAGGTGTGTCCCAGGTGCTTAACCGATACACGTTTGCTTCGACCCTGTCACATTTG  240
                  ||||||||||||||||||||||||||||||||||||||||||||||||||||||||||||
HMAS248844  202   ACTGCAGGTGTGTCCCAGGTGCTTAACCGATACACGTTTGCTTCGACCCTGTCACATTTG  261

T069        241   CGTCGTACCAACACTCCCATCGGAAGAGATGGTAAGCTGGCAAAGCCTCGACAGCTTCAC  300
                  ||||||||||||||||||||||||||||||||||||||||||||||||||||||||||||
HMAS248844  262   CGTCGTACCAACACTCCCATCGGAAGAGATGGTAAGCTGGCAAAGCCTCGACAGCTTCAC  321

T069        301   AACACGCATTGGGGTTTGGTCTGCCCAGCCGAGACACCCGAAGGACAGGCCTGTGGTCTG  360
                  ||||||||||||||||||||||||||||||||||||||||||||||||||||||||||||
HMAS248844  322   AACACGCATTGGGGTTTGGTCTGCCCAGCCGAGACACCCGAAGGACAGGCCTGTGGTCTG  381

T069        361   GTCAAGAACCTGTCTTTGATGTGTTACGTCAGTGTCGGTTCTCCCTCCGAGCCTCTGATT  420
                  ||||||||||||||||||||||||||||||||||||||||||||||||||||||||||||
HMAS248844  382   GTCAAGAACCTGTCTTTGATGTGTTACGTCAGTGTCGGTTCTCCCTCCGAGCCTCTGATT  441

T069        421   GAGTTCATGATCAACAGAGGTATGGAAGTCGTCGAAGAGTATGAGCCTCTGCGGTATCCT  480
                  ||||||||||||||||||||||||||||||||||||||||||||||||||||||||||||
HMAS248844  442   GAGTTCATGATCAACAGAGGTATGGAAGTCGTCGAAGAGTATGAGCCTCTGCGGTATCCT  501

T069        481   CATGCTACAAAGATTTTTGTGAACGGTGTCTGGGTTGGAGTTCACCAAGACCCTAAGCAC  540
                  ||||||||||||||||||||||||||||||||||||||||||||||||||||||||||||
HMAS248844  502   CATGCTACAAAGATTTTTGTGAACGGTGTCTGGGTTGGAGTTCACCAAGACCCTAAGCAC  561

T069        541   TTGGTGAACCAGGTTCTAGATACTCGTCGCAAGTCCTATCTGCAATACGAAGTCTCTCTC  600
                  ||||||||||||||||||||||||||||||||||||||||||||||||||||||||||||
HMAS248844  562   TTGGTGAACCAGGTTCTAGATACTCGTCGCAAGTCCTATCTGCAATACGAAGTCTCTCTC  621

T069        601   GTGAGAGAAATTCGAGACCAGGAATTCAAAATCTTTTCCGACGCAGGCCGTGTCATGCGA  660
                  ||||||||||||||||||||||||||||||||||||||||||||||||||||||||||||
HMAS248844  622   GTGAGAGAAATTCGAGACCAGGAATTCAAAATCTTTTCCGACGCAGGCCGTGTCATGCGA  681

T069        661   CCAGTCTTTACCGTTCAGCAGGAAGATGACCCAGAAACGGGCATCAACAAGGGTCACCTG  720
                  |||||||||||||||||||||||||||||||| |||||||||||||||||||||||||||
HMAS248844  682   CCAGTCTTTACCGTTCAGCAGGAAGATGACCCGGAAACGGGCATCAACAAGGGTCACCTG  741

T069        721   GTATTGACCAAGGAGCTCGTCAATAGATTGGCCAAGGAGCAGGCTGAGCCTCCGGAAGAT  780
                  ||||||||||||||||||||||||||||||||||||||||||||||||||||||||||||
HMAS248844  742   GTATTGACCAAGGAGCTCGTCAATAGATTGGCCAAGGAGCAGGCTGAGCCTCCGGAAGAT  801

T069        781   CCCAGCATGAAGATCGGATGGGAGGGATTAATCAGGGCTGGTGCGGTTGAATATCTCGAC  840
                  ||||||||||||||||||||||||||||||||||||||||||||||||||||||||||||
HMAS248844  802   CCCAGCATGAAGATCGGATGGGAGGGATTAATCAGGGCTGGTGCGGTTGAATATCTCGAC  861

T069        841   GCCGAGGAAGAGGAGACGTCCATGATCTGCATGACGCCAGAGGATCTCGAGTTGTATCGT  900
                  ||||||||||||||||||||||||||||||||||||||||||||||||||||||||||||
HMAS248844  862   GCCGAGGAAGAGGAGACGTCCATGATCTGCATGACGCCAGAGGATCTCGAGTTGTATCGT  921

T069        901   CTTCAGAAGGCCGGTATTAACACTGAGGAAGACATGGGAGATGACCCGAACAAGCGACTA  960
                  ||||||||||||||||||||||||||||||||||||||||||||||||||||||||||||
HMAS248844  922   CTTCAGAAGGCCGGTATTAACACTGAGGAAGACATGGGAGATGACCCGAACAAGCGACTA  981

T069        961   AAGACCAAGACCAACCCGACAACTCACATGTACACCCATTGCGAGATTCACCCAAGTATG  1020
                  ||||||||||||||||||||||||||||||||||||||||||||||||||||||||||||
HMAS248844  982   AAGACCAAGACCAACCCGACAACTCACATGTACACCCATTGCGAGATTCACCCAAGTATG  1041

T069        1021  ATCTTAGGCATCTGTGCTAGTATCATTCCTTTCCCCGATCACAAC  1065
                  |||||||||||||||||||||||||||||||||||||||||||||
HMAS248844  1042  ATCTTAGGCATCTGTGCTAGTATCATTCCTTTCCCCGATCACAAC  1086
```

Fig. S1. Sequence alignment between *T. breve* T069 and *T. breve* voucher HMAS:248844 for *rpb2*.

Score	Expect	Identities	Gaps	Strand
1417 bits(767)	0.0	767/767(100%)	0/767(0%)	Plus/Plus

```
T069        1     AGGCTGACTGCGCCATTCTCATCATTGCCGCCGGTACTGGTGAGTTCGAGGCTGGTATCT  60
                  ||||||||||||||||||||||||||||||||||||||||||||||||||||||||||||
HMAS248844  348   AGGCTGACTGCGCCATTCTCATCATTGCCGCCGGTACTGGTGAGTTCGAGGCTGGTATCT  407

T069        61    CCAAGGATGGCCAGACTCGTGAGCACGCTCTGCTCGCCTACACCCTGGGTGTCAAGCAGC  120
                  ||||||||||||||||||||||||||||||||||||||||||||||||||||||||||||
HMAS248844  408   CCAAGGATGGCCAGACTCGTGAGCACGCTCTGCTCGCCTACACCCTGGGTGTCAAGCAGC  467

T069        121   TTATCGTTGCCATCAACAAGATGGACACTGCCAACTGGGCCGAGGCTCGTTACCAGGAAA  180
                  ||||||||||||||||||||||||||||||||||||||||||||||||||||||||||||
HMAS248844  468   TTATCGTTGCCATCAACAAGATGGACACTGCCAACTGGGCCGAGGCTCGTTACCAGGAAA  527

T069        181   TCATCAAGGAGACTTCCAACTTCATCAAGAAGGTCGGCTTCAACCCCAAGGCTGTTGCTT  240
                  ||||||||||||||||||||||||||||||||||||||||||||||||||||||||||||
HMAS248844  528   TCATCAAGGAGACTTCCAACTTCATCAAGAAGGTCGGCTTCAACCCCAAGGCTGTTGCTT  587

T069        241   TCGTCCCCATCTCCGGTTTCAACGGTGACAACATGCTCCAGCCCTCCACCAACTGCCCCT  300
                  ||||||||||||||||||||||||||||||||||||||||||||||||||||||||||||
HMAS248844  588   TCGTCCCCATCTCCGGTTTCAACGGTGACAACATGCTCCAGCCCTCCACCAACTGCCCCT  647

T069        301   GGTACAAGGGTTGGGAGAAGGAGACCAAGGCTGGCAAGTTCACCGGCAAGACCCTCCTTG  360
                  ||||||||||||||||||||||||||||||||||||||||||||||||||||||||||||
HMAS248844  648   GGTACAAGGGTTGGGAGAAGGAGACCAAGGCTGGCAAGTTCACCGGCAAGACCCTCCTTG  707

T069        361   AGGCCATCGACTCCATCGAGCCCCCCAAGCGTCCCACGGACAAGCCCCTCCGTCTTCCCC  420
                  ||||||||||||||||||||||||||||||||||||||||||||||||||||||||||||
HMAS248844  708   AGGCCATCGACTCCATCGAGCCCCCCAAGCGTCCCACGGACAAGCCCCTCCGTCTTCCCC  767

T069        421   TCCAGGATGTCTACAAGATCGGTGGTATCGGAACAGTTCCCGTCGGCCGTATCGAGACTG  480
                  ||||||||||||||||||||||||||||||||||||||||||||||||||||||||||||
HMAS248844  768   TCCAGGATGTCTACAAGATCGGTGGTATCGGAACAGTTCCCGTCGGCCGTATCGAGACTG  827

T069        481   GTGTCCTCAAGCCCGGTATGGTCGTCACCTTCGCTCCCTCCAACGTCACCACTGAAGTCA  540
                  ||||||||||||||||||||||||||||||||||||||||||||||||||||||||||||
HMAS248844  828   GTGTCCTCAAGCCCGGTATGGTCGTCACCTTCGCTCCCTCCAACGTCACCACTGAAGTCA  887

T069        541   AGTCCGTCGAGATGCACCACGAGCAGCTCACCGAGGGTGTTCCCGGTGACAACGTTGGTT  600
                  ||||||||||||||||||||||||||||||||||||||||||||||||||||||||||||
HMAS248844  888   AGTCCGTCGAGATGCACCACGAGCAGCTCACCGAGGGTGTTCCCGGTGACAACGTTGGTT  947

T069        601   TCAACGTCAAGAACGTTTCCGTTAAGGAAATTCGCCGTGGTAACGTTGCCGGTGACTCCA  660
                  ||||||||||||||||||||||||||||||||||||||||||||||||||||||||||||
HMAS248844  948   TCAACGTCAAGAACGTTTCCGTTAAGGAAATTCGCCGTGGTAACGTTGCCGGTGACTCCA  1007

T069        661   AGAACGAcccccccATGGGTGCCGCTTCTTTCACCGCTCAGGTCATCGTCATGAACCACC  720
                  ||||||||||||||||||||||||||||||||||||||||||||||||||||||||||||
HMAS248844  1008  AGAACGACCCCCCCATGGGTGCCGCTTCTTTCACCGCTCAGGTCATCGTCATGAACCACC  1067

T069        721   CTGGCCAGGTCGGTGCCGGCTACGCCCCCGTTCTTGACTGCCACACT  767
                  |||||||||||||||||||||||||||||||||||||||||||||||
HMAS248844  1068  CTGGCCAGGTCGGTGCCGGCTACGCCCCCGTTCTTGACTGCCACACT  1114
```

Fig. S2. Sequence alignment between *T. breve* T069 and *T. breve* voucher HMAS:248844 for *tef1*.

Score	Expect	Identities	Gaps	Strand
1038 bits(562)	0.0	567/569(99%)	1/569(0%)	Plus/Plus

```
T069        33   CCGAGTTTACAACTCCCAAACCCAATGTGAACGTTACCAAACTGTTGCCTCGGCGGGATC  92
                 ||||||||||||||||||||||||||||||||||||||||||||||||||||||||||||
HMAS248844  1    CCGAGTTTACAACTCCCAAACCCAATGTGAACGTTACCAAACTGTTGCCTCGGCGGGATC  60

T069        93   TCTGCCCCGGGTGCGTCGCAGCCCCGGACCAAGGCGCCCGCCGGAGGACCAACCAAAACT  152
                 ||||||||||||||||||||||||||||||||||||||||||||||||||||||||||||
HMAS248844  61   TCTGCCCCGGGTGCGTCGCAGCCCCGGACCAAGGCGCCCGCCGGAGGACCAACCAAAACT  120

T069        153  CTTTTTGTATACCCCCTCGCGGGTTTTTT-ATAATCTGAGCCTTCTCGGCGCCTCTCGTA  211
                 ||| ||||||||||||||||||||||||| ||||||||||||||||||||||||||||||
HMAS248844  121  CTTATTGTATACCCCCTCGCGGGTTTTTTAATAATCTGAGCCTTCTCGGCGCCTCTCGTA  180

T069        212  GGCGTTTCGAAAATGAATCAAAACTTTCAACAACGGATCTCTTGGTTCTGGCATCGATGA  271
                 ||||||||||||||||||||||||||||||||||||||||||||||||||||||||||||
HMAS248844  181  GGCGTTTCGAAAATGAATCAAAACTTTCAACAACGGATCTCTTGGTTCTGGCATCGATGA  240

T069        272  AGAACGCAGCGAAATGCGATAAGTAATGTGAATTGCAGAATTCAGTGAATCATCGAATCT  331
                 ||||||||||||||||||||||||||||||||||||||||||||||||||||||||||||
HMAS248844  241  AGAACGCAGCGAAATGCGATAAGTAATGTGAATTGCAGAATTCAGTGAATCATCGAATCT  300

T069        332  TTGAACGCACATTGCGCCCGCCAGTATTCTGGCGGGCATGCCTGTCCGAGCGTCATTTCA  391
                 ||||||||||||||||||||||||||||||||||||||||||||||||||||||||||||
HMAS248844  301  TTGAACGCACATTGCGCCCGCCAGTATTCTGGCGGGCATGCCTGTCCGAGCGTCATTTCA  360

T069        392  ACCCTCGAACCCCTCCGGGGGGTCGGCGTTGGGGATCGGCCCTCCCTTAGCGGGTGGCCG  451
                 ||||||||||||||||||||||||||||||||||||||||||||||||||||||||||||
HMAS248844  361  ACCCTCGAACCCCTCCGGGGGGTCGGCGTTGGGGATCGGCCCTCCCTTAGCGGGTGGCCG  420

T069        452  TCTCCGAAATACAGTGGCGGTCTCGCCGCAGCCTCTCCTGCGCAGTAGTTTGCACACTCG  511
                 ||||||||||||||||||||||||||||||||||||||||||||||||||||||||||||
HMAS248844  421  TCTCCGAAATACAGTGGCGGTCTCGCCGCAGCCTCTCCTGCGCAGTAGTTTGCACACTCG  480

T069        512  CATCGGGAGCGCGGCGCGTCCACAGCCGTTAAACACCCAACTTCTGAAATGTTGACCTCG  571
                 ||||||||||||||||||||||||||||||||||||||||||||||||||||||||||||
HMAS248844  481  CATCGGGAGCGCGGCGCGTCCACAGCCGTTAAACACCCAACTTCTGAAATGTTGACCTCG  540

T069        572  GATCAGGTAGGAATACCCGCTGAACTTAA  600
                 |||||||||||||||||||||||||||||
HMAS248844  541  GATCAGGTAGGAATACCCGCTGAACTTAA  569
```

Fig. S3. Sequence alignment between *T. breve* T069 and *T. breve* voucher HMAS:248844 for ITS.

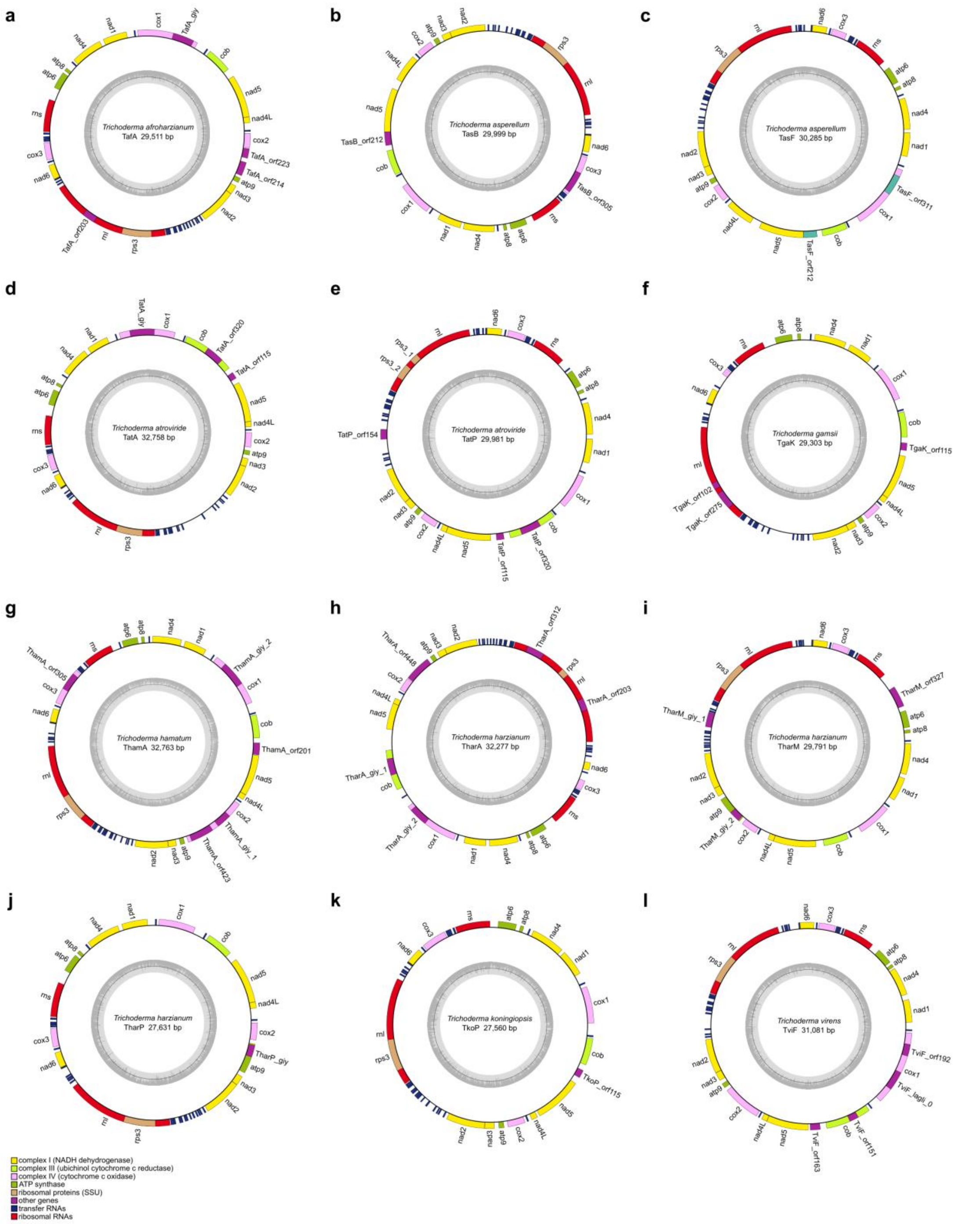

Fig. S4. Mitogenome map of *T. afroharzianum* TafA (a), *T. asperellum* TasB (b), *T. asperellum* TasF (c), *T. atroviride* TatA (d), *T. atroviride* TatP (e), *T. gamsii* TgaK (f), *T. hamatum* ThamA (g), *T. harzianum* TharA (h), *T. harzianum* TharM (i), *T. harzianum* TharP (j), *T. koningiopsis* TkoP (k), and *T. virens* TviF (l).

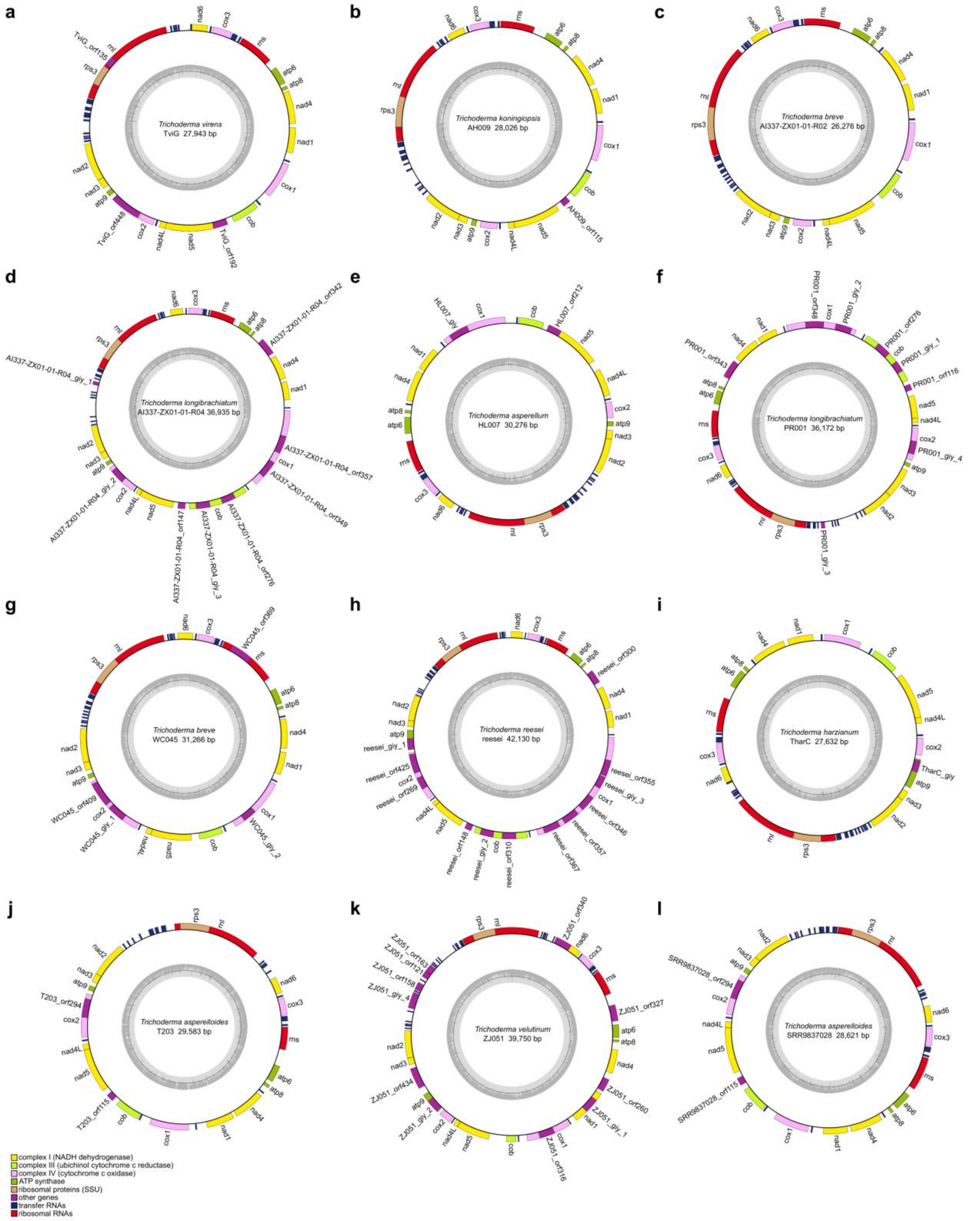

Fig. S5. Mitogenome map of *T. virens* TviG (a), *T. koningiopsis* AH009 (b), *T. breve* AI337-ZX01-01-R02 (c), *T. longibrachiatum* AI337-ZX01-01-R04 (d), *T. asperellum* HL007 (e), *T. longibrachiatum* PR001 (f), *T. breve* WC045 (g), *T. reesei* reesei (h), *T. harzianum* TharC (i), *T. asperelloides* T203 (j), *T. velutinum* ZJ051 (k), and *T. asperelloides* SRR9837028 (l).

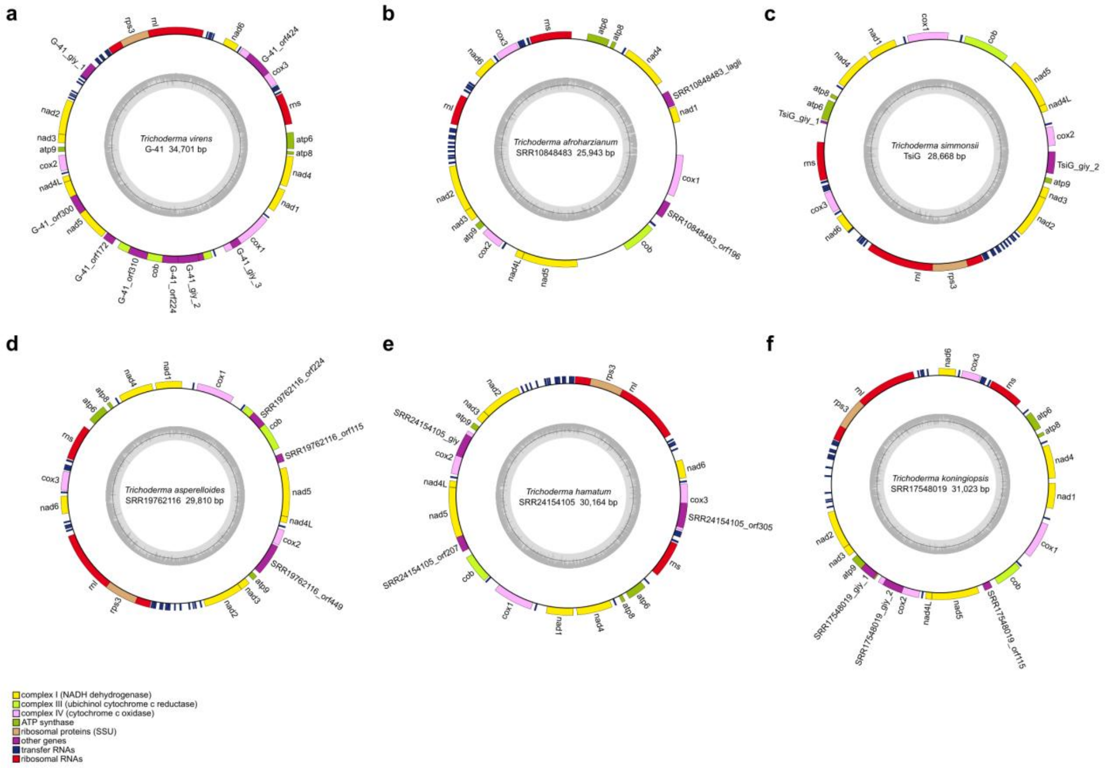

Fig. S6. Mitogenome map of *T. virens* G-41 (a), *T. afroharzianum* SRR10848483 (b), *T. simmonsii* TsiG (c), *T. asperelloides* SRR19762116 (d), *T. hamatum* SRR24154105 (e), and *T. koningiopsis* SRR17548019 (f).

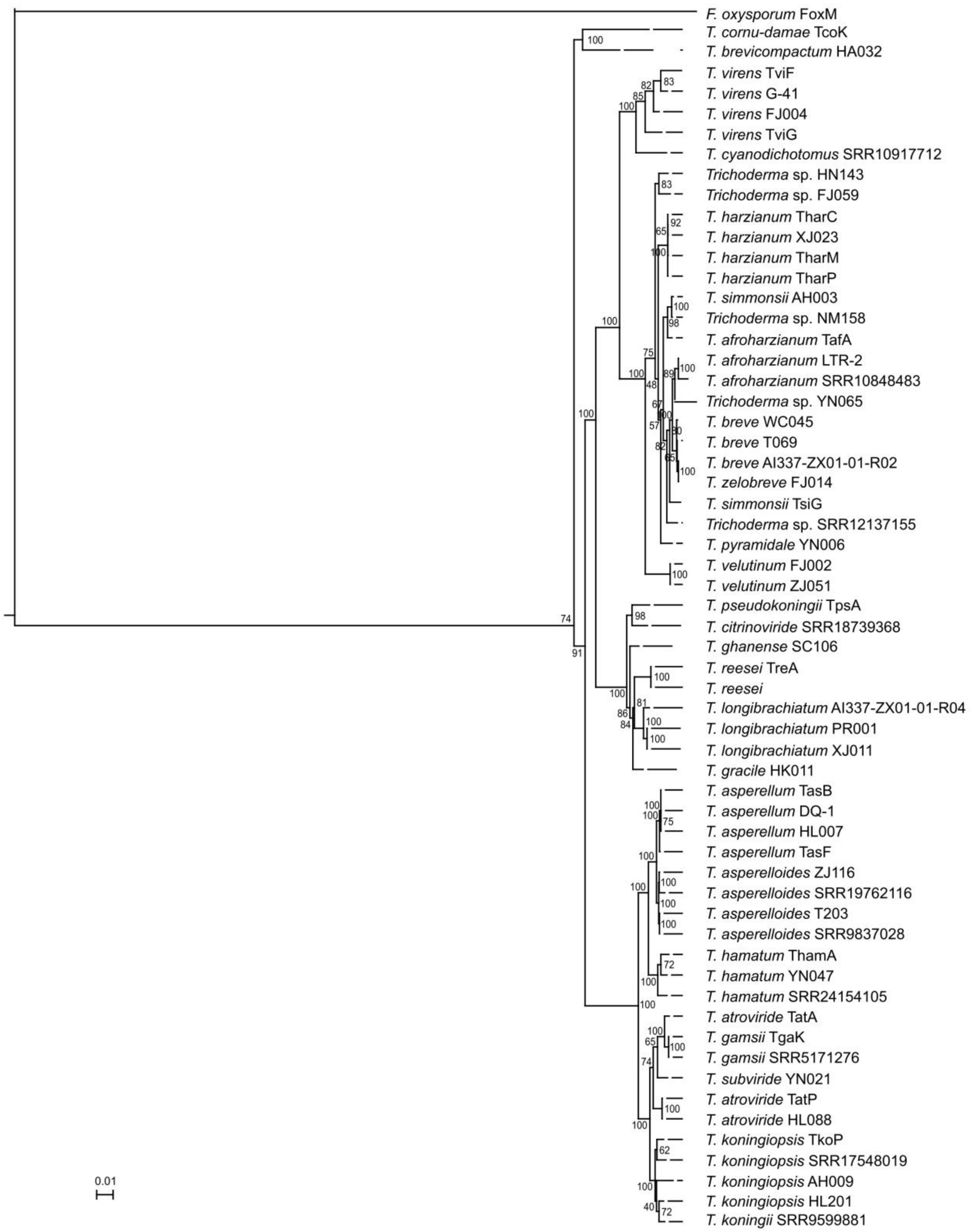

Fig. S7. Phylogenetic tree inferred from the whole mitogenome sequences of 59 *Trichoderma* strains, based on Maximum likelihood (ML) methods. The best model of GTR+I+G with bootstrap value of 1000 replicates was used to construct the phylogeny, and the *Fusarium oxysporum* mh2-2 was used as outgroup.

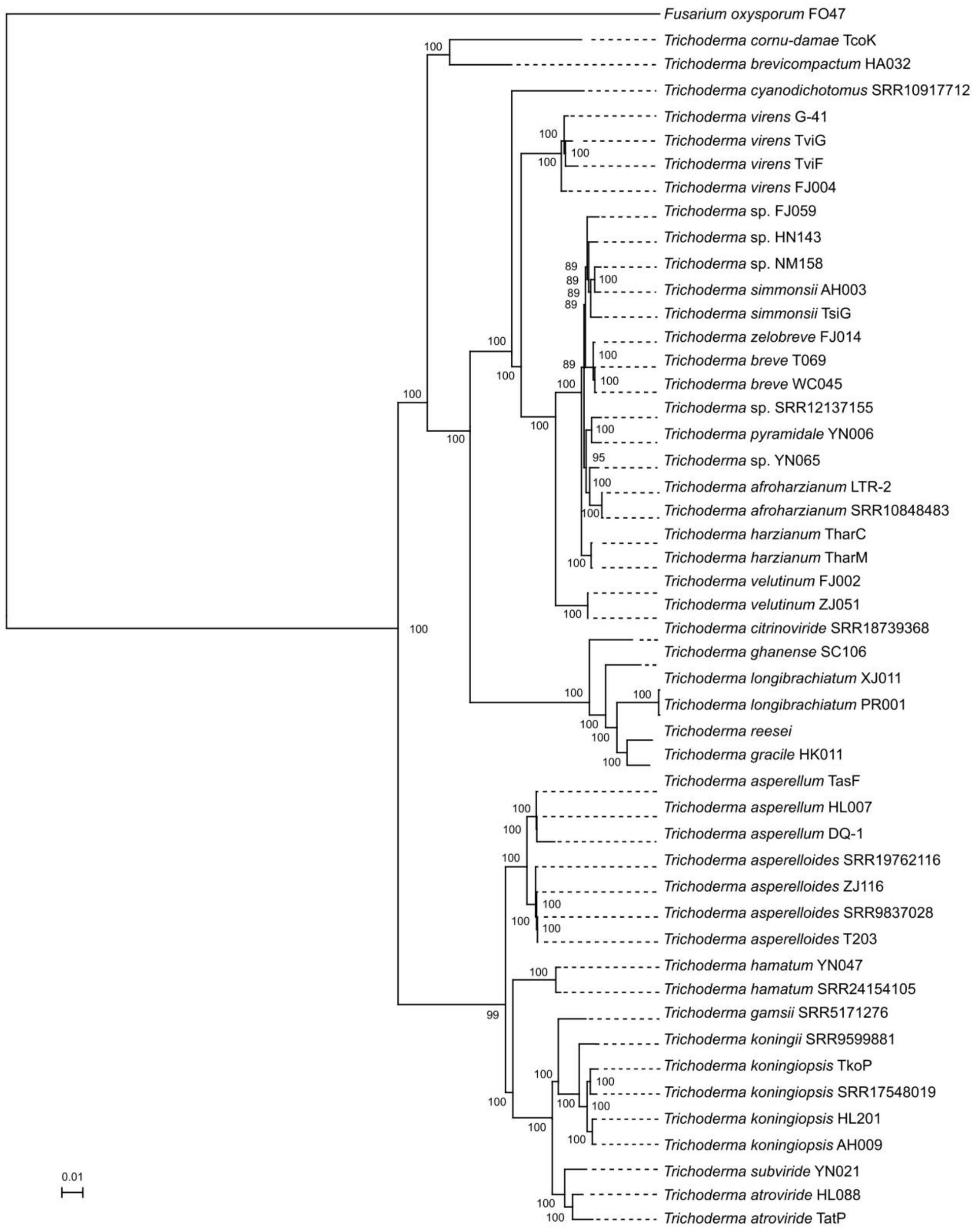

Fig. S8. Phylogenetic tree inferred from the amino acid sequences of nuclear genes from 48 *Trichoderma* genomes, based on Maximum likelihood (ML) methods. The best model of JTT+I+G+F with bootstrap value of 500 replicates was used to construct the phylogeny, and the *F. oxysporum* FO47 was used as outgroup.

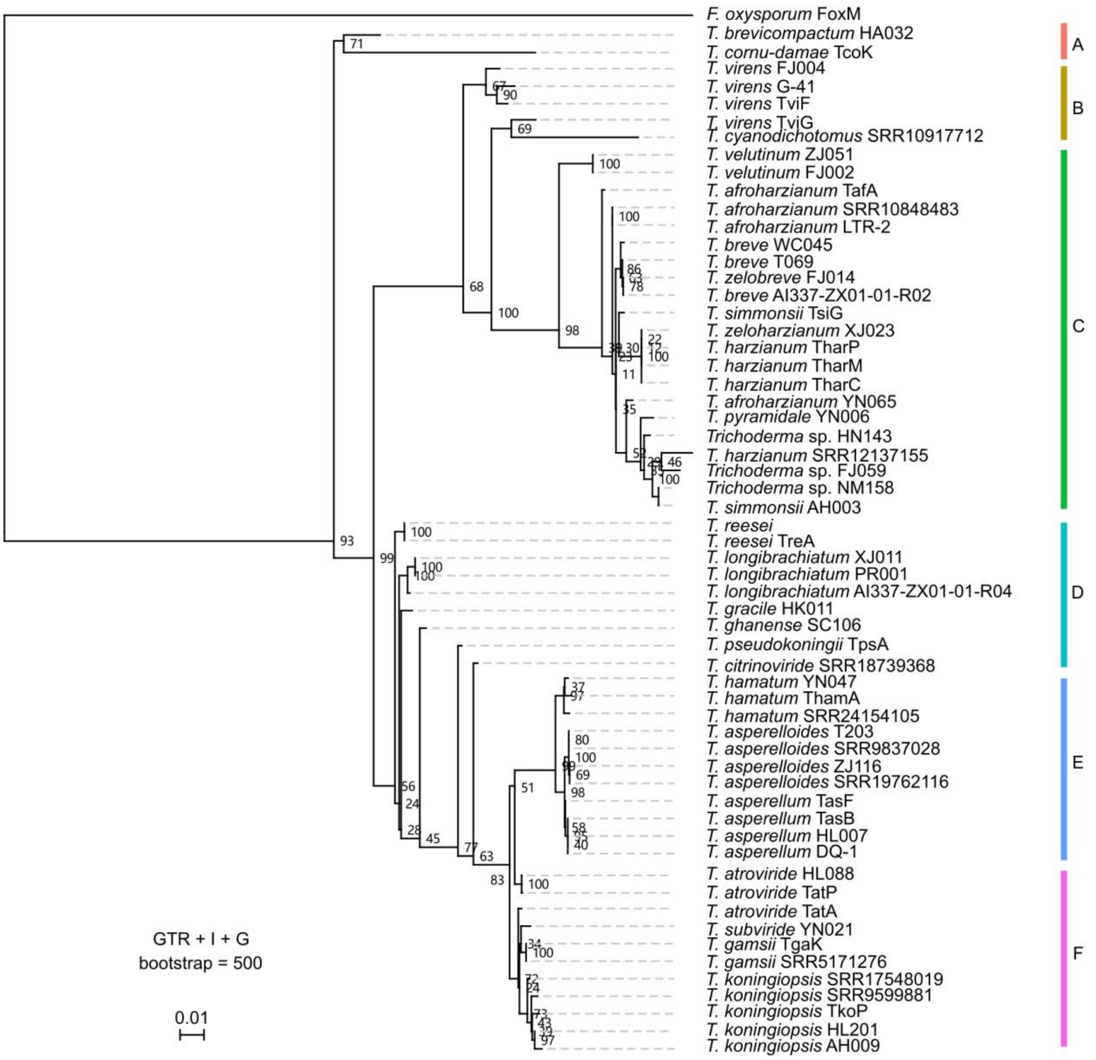

Fig. S9. Phylogenetic tree inferred from concatenation of sequences from *nad3*, *cox2*, and *nad5* genes of 59 *Trichoderma* strains, based on Maximum likelihood (ML) methods. The best model of GTR+I+G with bootstrap value of 500 replicates was used to construct the phylogeny, and the *F. oxysporum* mh2-2 was used as outgroup.
